# Immune-mediated indirect interaction between gut microbiota and bacterial pathogens

**DOI:** 10.1186/s12915-025-02399-1

**Published:** 2025-09-16

**Authors:** Maryam Keshavarz, Mathias Franz, Haicheng Xie, Caroline Zanchi, Susan Mbedi, Sarah Sparmann, Jens Rolff

**Affiliations:** 1 Evolutionary Biology, Institute of Biology, Freie Universität Berlin, Berlin, Germany; 2 Berlin Center for Genomics in Biodiversity Research, BeGenDiv, Berlin, Germany; 3 Museum Für Naturkunde, Berlin, Germany; 4 Leibniz-Institut Für Gewässerökologie Und Binnenfischerei (IGB), Berlin, Germany

**Keywords:** Host–pathogen-microbiota interactions, Gut microbiota, Imd-dependent antimicrobial peptides, Relish, *Tenebrio molitor*

## Abstract

**Background:**

In many animals, survival during infection depends on the ability to coordinate interactions between the host immune system and gut microbiota. These tripartite interactions, in turn, potentially shape pathogen virulence evolution. A key regulator of the immune system and, hence, bipartite interactions in insects is the immune deficiency (Imd) pathway, which modulates gut microbiota and pathogens by synthesizing antimicrobial peptides (AMPs) through the NF-κB transcription factor *Relish*. However, whether Imd-dependent AMPs mediate indirect interactions between gut microbiota and pathogens in a tripartite context remains unclear. Using RNAi-mediated knockdown of *Tenebrio molitor Relish* (*TmRelish*), we hypothesized that Imd-dependent AMPs influence indirect interaction between *Providencia burhodogranariea_B* (*P. b_B*) infection and the gut microbiota.

**Results:**

*TmRelish* knockdown altered bipartite interactions by disrupting gut microbiota load and composition, increasing pathogen load, and ultimately leading to higher host mortality during infection. However, we did not find support for our tripartite hypothesis that Imd-dependent AMPs mediate indirect interactions between the gut microbiota and *P. b_B* infection, suggesting the involvement of alternative regulatory pathways or Imd-independent mechanisms. Nevertheless, our investigations of tripartite interactions showed a positive effect of *P. b_B* infection on gut microbiota load, which in turn stimulated the expression of a subset of AMPs. However, this upregulation of AMPs did not result in reduced *P. b_B* load. Notably, the gut microbiota did not affect pathogen load but promoted host survival during *P. b_B* infection, indicating a role in increasing host tolerance rather than resistance.

**Conclusions:**

These findings suggest that while Imd-dependent AMPs may not mediate tripartite interactions in our system, microbiota-host interactions, such as microbiota-mediated immune priming and changes in microbiota load, can shape infection outcomes. These effects on infection outcomes almost certainly exert important selective pressures on the evolution of bacterial virulence.

**Supplementary Information:**

The online version contains supplementary material available at 10.1186/s12915-025-02399-1.

## Background

Pathogen virulence and its evolution can depend on the presence of host gut microbiota [1–3]. The gut microbiota, however, can be a double-edged sword [4, 5], by either mediating protection [6–8], or facilitating infection of the host by invading pathogens [9, 10] with opposite outcomes for host survival. Also, the metabolic [11, 12] and immunological environments [13, 14] are key contributors to the pathogenicity of invaders. These tripartite interactions between host, its microbiota, and pathogens have so far gained little attention in invertebrates. One of the few studies showed in *Anopheles stephensi* that fungal infection by *Beauveria bassiana* downregulated gut dual oxidase (Duox) and antimicrobial peptides (AMPs), leading to the overgrowth and translocation of the opportunistic midgut bacterium *Serratia marcescens* from the gut to the hemocoel, ultimately increasing host mortality [15]. Another example is found in the Pacific oyster, *Crassostrea gigas*, where viral infection of hemocytes by *Ostreid herpesvirus* impaired AMP expression, leading to a significant increase in the load of rare gut microbiota, such as *Vibrio* and *Arcobacter*, promoting host death [16]. Thus, in both cases, pathogen infections downregulate AMP-mediated host defenses, leading to alterations in gut microbiota load, suggesting that pathogens and gut microbiota interact indirectly through immune-mediated mechanisms. 

Host immune-mediated indirect interactions between gut microbiota and pathogens could appear in different forms, based on how the microbiota and pathogens influence, or are influenced by, the host immune system [1]. Depending on the balance between immune activation and suppression, host–pathogen-microbiota interactions can be negatively or positively modified and classified as apparent competition, indirect amensalism, or apparent mutualism [17]. In apparent competition, the negative effect could be symmetric, for example, when host immunity equally suppresses both microbiota and pathogens, or asymmetric when one is more strongly suppressed than the other. Indirect amensalism occurs when host immunity exerts a disproportionately negative effect on pathogen infection while allowing gut microbiota to thrive, thereby favoring the microbiota over the pathogen or vice versa. In contrast, apparent mutualism between the pathogen and microbiota arises when host immunity has a positive effect, facilitating the proliferation of both microbiota and pathogens. Ultimately, the nature of these immune-mediated interactions imposes selection pressures on pathogen traits, potentially shaping virulence evolution [1, 17, 18]. Predictions for different forms of interactions in virulence evolution depend on such tripartite interactions [1, 17].

Empirical support for indirect interactions comes from earlier studies in mosquitoes, flies, and oysters. For example, in *Aedes aegypti*, dengue virus infection is suppressed by the gut microbiota through basal level activation of Toll pathway, a case of apparent competition [19]. A comparable mechanism occurs in *Drosophila melanogaster*, where the symbiont *Wolbachia* enhances resistance to RNA viruses through immune activation [20]. In contrast, in *Drosophila*, the commensal *Lactiplantibacillus plantarum* shows resilience during oral infection with the Gram-negative pathogen *Erwinia carotovora carotovora 15* (*Ecc15*) due to its resistance to host AMPs. *Ecc15* infection activates Imd-dependent AMPs that suppress the pathogen while sparing *L. plantarum*, indicating indirect amensalism [21]. In *A. stephensi*, fungal infection by *B. bassiana* perturbs the gut microbiota homeostasis, leading to an increase in bacterial load that accelerates host mortality, suggesting an apparent mutualism between the pathogen and gut microbiota [15]. A similar disruption occurs in Pacific oysters, *Crassostrea gigas*, where OsHV-1 µVar infection impairs AMP expression, leading to proliferation of opportunistic microbiota [16].

Host immunity mediates tolerance toward gut microbiota (host-microbiota) while providing resistance against pathogen infections (host–pathogen) through different mechanisms, including antimicrobial activity. One of the well-known innate immune signaling pathways in insects is the Imd pathway, which is considered a master regulator of gut microbiota and a key defense mechanism against pathogenic infections [22, 23]. Studies in *Drosophila*, for example, have demonstrated that local gut Imd-induced immunity confronts ingested microorganisms through synthesizing a cocktail of effector molecules, including AMPs, thereby regulating microbial community dynamics and host interactions [24, 25]. One of the key components of Imd pathway is the NF-κB transcription factor *Relish* that translocates into the nucleus to activate AMP expression [26]. This is supported by studies in *Relish*-deficient insects, such as *Drosophila* [21, 22, 27], the red palm weevil (*Rhynchophorus ferrugineus*) [28], and the oriental fruit fly *Bactrocera dorsalis* [29], in which disruption of AMP expression alters both the composition and bacterial loads of gut-associated microbiota. While several studies have focused on *Relish*-mediated responses following oral infections, there is also evidence that systemic infections with Gram-negative bacteria (e.g., *Escherichia coli*) can trigger local AMP expression in insects such as mealworm beetles, *Tenebrio molitor* and *R. ferrugineus* [28, 30]. Yet, alterations in gut microbiota are not solely driven by changes in AMP expression. Gut microbiota-induced reactive oxygen species (ROS) regulate Imd-mediated AMP expression, suggesting a crosstalk between Duox and the Imd pathway [27, 31]. In addition, there is compelling evidence in fruit flies [23, 32], mosquitoes [33, 34], and *T. molitor* [30] that *Relish* knockdown renders insects more susceptible to pathogenic infections. Five families of inducible antibacterial AMPs with multiple isoforms have been identified in *T. molitor*: Attacins [35], Tenecins [36], Coleoptericins [37], Defensins [38], and Cecropins [39]. Here, we investigate how Imd-dependent AMP expression modulates interactions between gut microbiota and pathogens in an insect host, using *T. molitor*, a reliable model for studying host–pathogen-microbiota interactions due to its well-characterized immune system and tractable gut microbiota [40, 41]. Given the importance of *Relish*-mediated AMPs in bipartite interactions between host-microbiota and host–pathogen, we hypothesize that this pathway also plays a role in shaping indirect interactions (with positive or negative effect) between pathogens and gut microbiota. In the present work, we used RNAi-mediated knockdown of *Relish* to generate different transcriptional levels of AMPs [30]. As a bacterial model, we used *Providencia burhodogranariea_B* (*P. b_B*), a Gram-negative bacterium isolated from the hemolymph of wild *Drosophila melanogaster* [42], which previously has been shown to cause increased mortality in infected flies, indicating relatively high virulence [43].

**Fig. 1:**
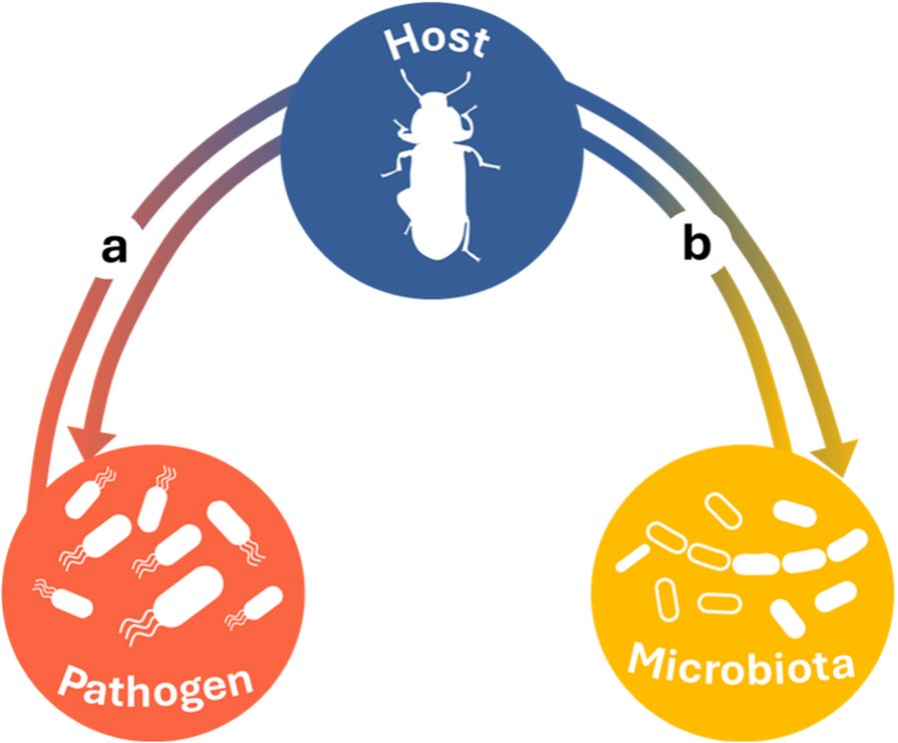
Indirect interaction between gut microbiota and pathogen via the host immune system. (**a**) Pathogenic infection affects the gut microbiota prevalence due to its effect on the host immune response. (**b**) The gut microbiota affects pathogenic infection outcomes due to its effect on the host immune response

**Fig. 2:**
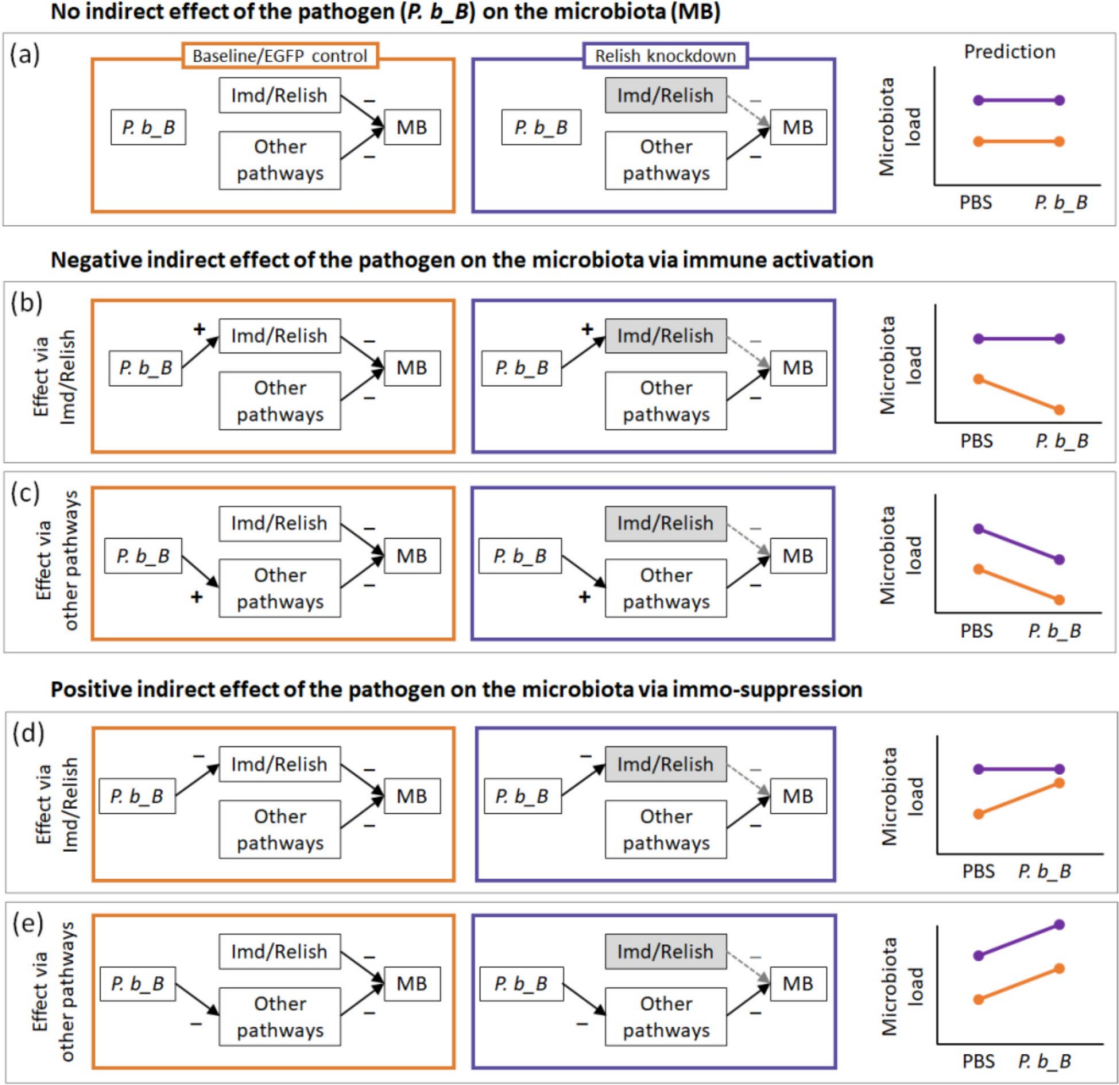
Conceptual predictions for indirect effects of *Providencia burhodogranariea_B* (*P. b_B*) infection on gut microbiota load. In all depicted scenarios, we assumed that gut microbiota is regulated by Imd-dependent antimicrobial peptides (AMPs) (via *Relish*) and other immune pathways, such that immune activation exerts a negative effect on microbiota load. Orange boxes and arrows represent baseline (ds*EGFP* control) conditions, whereas purple indicates *Relish* knockdown conditions, in which Imd pathway is assumed to be fully silenced. (**a**) Baseline scenario in which the pathogen exerts no indirect effect on gut microbiota. (**b**, **c**) Scenarios with a negative indirect effect (apparent competition) of the pathogen on microbiota through immune activation. (**d**, **e**) Scenarios with a positive indirect effect (apparent mutualism) of the pathogen on microbiota via immunosuppression. (**b**, **d**) In these scenarios, the indirect effect is mediated by Imd-dependent AMP expression. (c, e) In contrast, the indirect effect is mediated by other immune pathways independent of Imd-dependent AMP expression

To test our hypothesis, we first established how the bipartite interactions between the host and either its microbiota or a pathogen is influenced by Imd-mediated AMP expression in our model system. We then investigated whether and which form of indirect interactions occur in tripartite interaction context, and whether these indirect interactions are mediated by Imd-dependent AMP expression. Firstly, we studied the impact of pathogen infection on gut microbiota (Fig. 1a) and predicted that the effect of pathogen infection on gut microbiota load should be attenuated or absent in *Relish*-knockdown larvae (P1, Fig. 2c, d). Secondly, we studied the influence of gut microbiota on pathogen infection (Fig. 1b) and predicted also that the influence of gut microbiota on pathogen load should be absent or reduced in *Relish*-knockdown larvae (P2, Additional file 1: Fig. S1c, d). In addition, we investigated how the gut microbiota affects host survival during infection.

## Results

### Bipartite interaction between host and gut microbiota

**Fig. 3:**
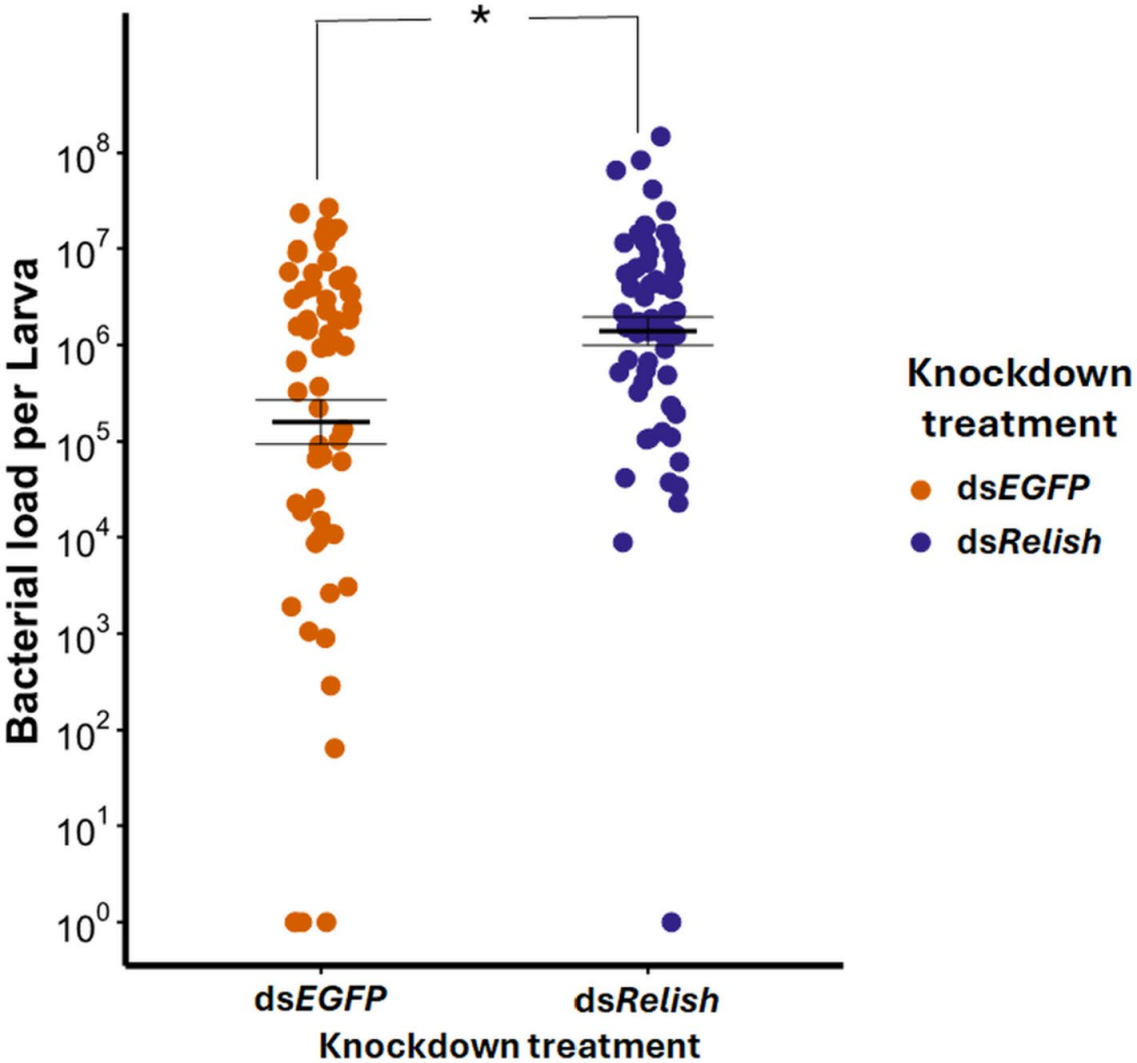
Bacterial load in larvae treated with ds*EGFP* and ds*TmRelish*. Bacterial load of all culturable bacteria was quantified using trypticase soy agar (TSA) plates. Means and standard errors are represented by the black lines, while the dots represent the biological replicates from individual guts (*n* = ∼12 per treatment, six independent experiments). Asterisk indicates significant difference between *TmRelish* knockdown and *EGFP* control (*z* = 3.461, *p* < 0.001)

We found that the knockdown of *TmRelish* led to a significant increase in microbial load, as demonstrated by the higher CFUs in ds*TmRelish*-treated larvae compared to the ds*EGFP* group (*z* = 3.461, *p* < 0.001) (Fig. 3). Additionally, *TmRelish* knockdown also altered the gut microbiota composition. Bray–Curtis dissimilarity analysis revealed a significant effect of knockdown (*R*^2^ = 0.29, *F* = 8.52, *p* < 0.001). Although the total microbial load increased, three bacterial genera, including *Bacillus* (*Bacillaceae 1* and *Bacillaceae 2*) and *Pediococcus* (*Lactobacillaceae*), were significantly reduced in abundance, suggesting a regulatory role of *TmRelish* in shaping the gut microbial community. Detailed taxonomic shifts and abundance data are provided in the supporting information (Additional file 1: Fig. S2 and Additional file 2: A).

### Bipartite interaction between host and pathogen

**Fig. 4:**
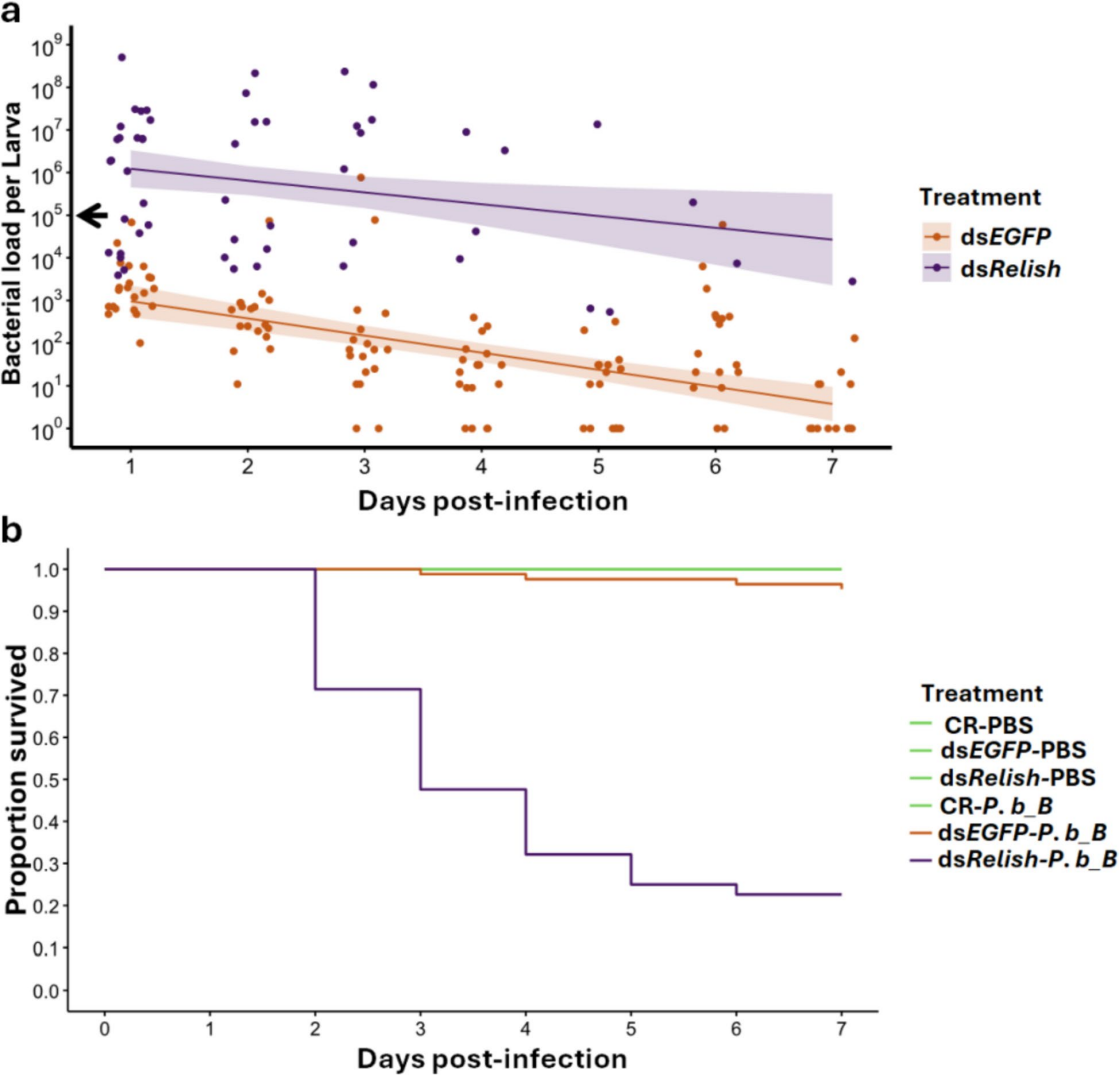
*Providencia burhodogranariea_B* load and host survival in *TmRelish*-knockdown larvae. **a** Bacterial load of *P. burhodogranariea_B* in ds*EGFP* and ds*TmRelish*-treated larvae over a period of 7 days (*n* = ∼10 per treatment per day, two independent experiments). The black arrow on the y-axis indicates the approximate injection dose. Purple and orange lines and associated bands depict the estimates and corresponding 95% confidence intervals of a linear model. Bacterial load was significantly higher in ds*TmRelish*-treated larvae compared to ds*EGFP* control (*F*_1, 176_ = 232.09, *p* < 0.001). **b** Survival of *T. molitor* larvae subjected to control (no dsRNA injection), ds*EGFP*, or ds*TmRelish* treatments, following exposure to either *P. burhodogranariea_B* (*P. b_B*) or PBS (*n* = ∼30 per treatment, three independent experiments), was monitored over a period of 7 days. No mortality was observed in larvae treated with PBS or in the CR-*P. b_B* groups (green), while *TmRelish*-knockdown (purple) larvae were short-lived compared to *EGFP* control (orange) (X.^2^_1, 18_ = 50.80, *p* < 0.001)

Next, we investigated whether *TmRelish* knockdown affects bacterial load, host survival, and expression levels of AMPs following infection. As expected, the *P. burhodogranariea_B* load was higher in ds*TmRelish* compared to ds*EGFP* larvae over the 7-day period (ds*EGFP*/ds*TmRelish*: *F*_1, 176_ = 232.09, *p* < 0.001) (Fig. 4a). Additionally, we found that the bacterial load in both ds*EGFP*- and ds*TmRelish*-treated larvae decreased over the 7-day period (time: *F*_1, 176_ = 59.30, *p* < 0.001) (Fig. 4a).

When we scored survival, no death was observed among larvae treated with phosphate buffered saline (PBS), as well as in the control-*P. b_B* groups (CR-PBS, ds*EGFP*-PBS, ds*TmRelish*-PBS, and CR-*P. b_B*). Therefore, these groups were excluded from further analysis, however, the corresponding data are presented in the figure (Fig. 4b). We found that more ds*TmRelish* larvae had died on day 7 compared to ds*EGFP* larvae (X^2^_1, 18_ = 50.80, *p* < 0.001) (Fig. 4b).

**Fig. 5:**
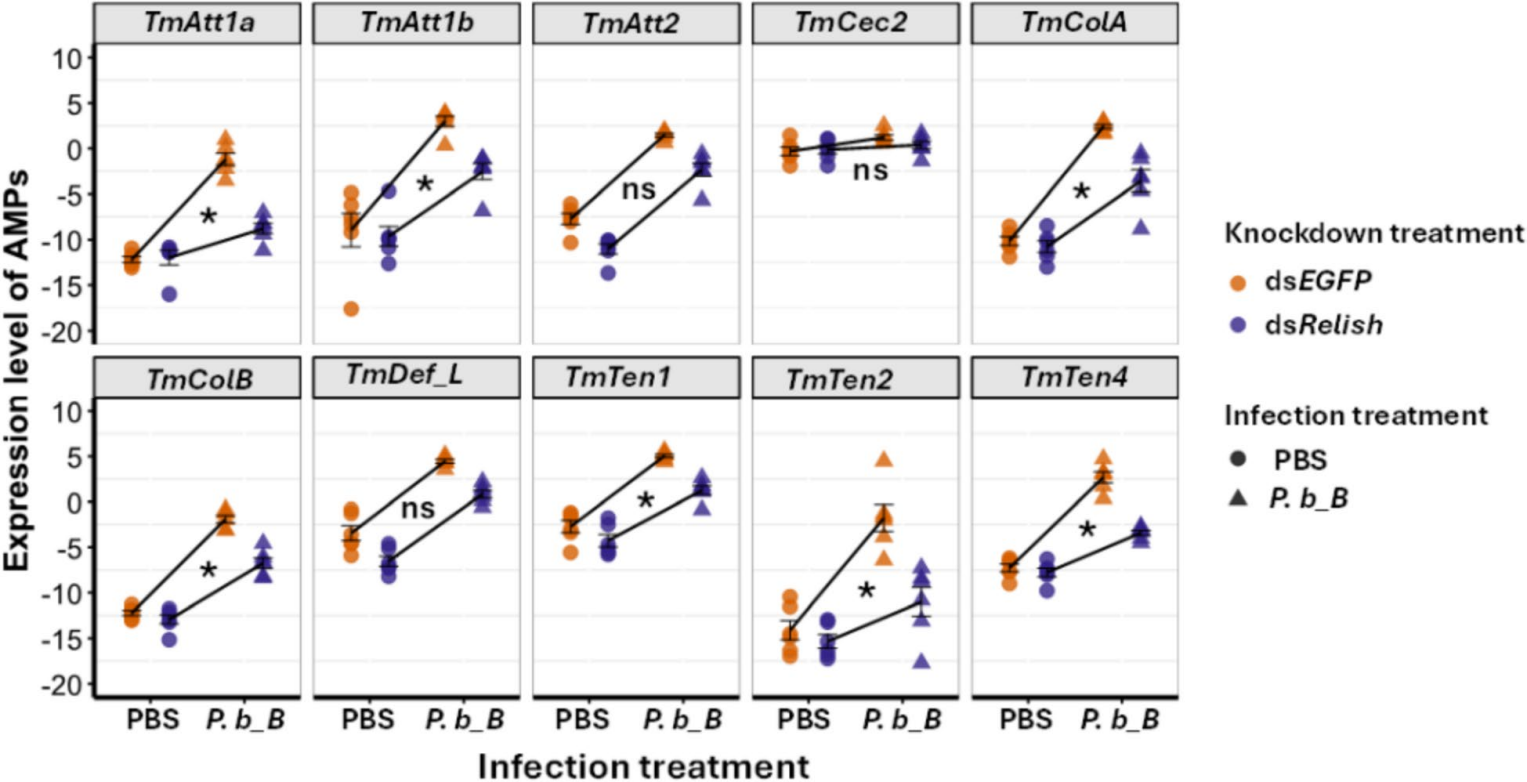
The effect of *Relish* knockdown on AMP expression post-infection. RT-qPCR analysis showing levels of *TmAtt1a*, *TmAtta1b*, *TmAtt2*, *TmTene1*, *TmTene2*, *TmTene4*, *TmColA*, *TmColB*, *TmCec2*, and *TmDef*-*L* mRNAs in whole-body extracts of ds*EGFP* (orange) and ds*TmRelish*-treated larvae (purple) infected with *P. burhodogranariea_B* (*P. b_B*, triangles) at 24 h post-infection. PBS-injected larvae (circles) were used as the mock control. *T. molitor 60S ribosomal protein L27a* (*TmL27a*) was used as the normalization control, using the delta Ct method (mean Ct of *TmL27a* − mean Ct of AMP gene). Data points represent individual biological replicates, and error bars denote the mean ± SEM. Asterisks indicate significant interaction between *TmRelish* knockdown and infection, ns, non-significant interaction

When investigating AMP expression, we found a significant interaction between knockdown treatment and infection on *TmAtt1a*, *TmAtt1b*, *TmTen1*, *TmTen2 TmTen4*, *TmColA*, and *TmColB* expression levels (Fig. 5, Additional file 1: Table S1). Infections generally led to an upregulation of these AMPs compared to non-infected PBS controls. However, for these seven AMPs, this upregulation was stronger in ds*EGFP*-treated controls compared to ds*TmRelish*-treated larvae (Fig. 5). This suggests that *TmRelish* at least partly caused infection-induced *AMP* upregulation, while its knockdown has little effect on basal AMP expression in non-infected larvae.

We did not find a significant interaction between knockdown treatment and infection on expression levels of *TmAtt2*, *TmDef_L*, and *TmCec2* (Fig. 5, Additional file 1: Table S1). These AMPs were upregulated following infection without any apparent influence of the knockdown treatment on the magnitude of this upregulation. While for *TmCec2*, there was no statistically significant effect of knockdown treatment, for *TmAtt2* and *TmDef_L*, we found a significant downregulation in knockdown larvae (independently of infection) (Fig. 5, Additional file 1: Table S1).

### The effect of pathogen infection on gut microbiota in tripartite interactions

**Fig. 6:**
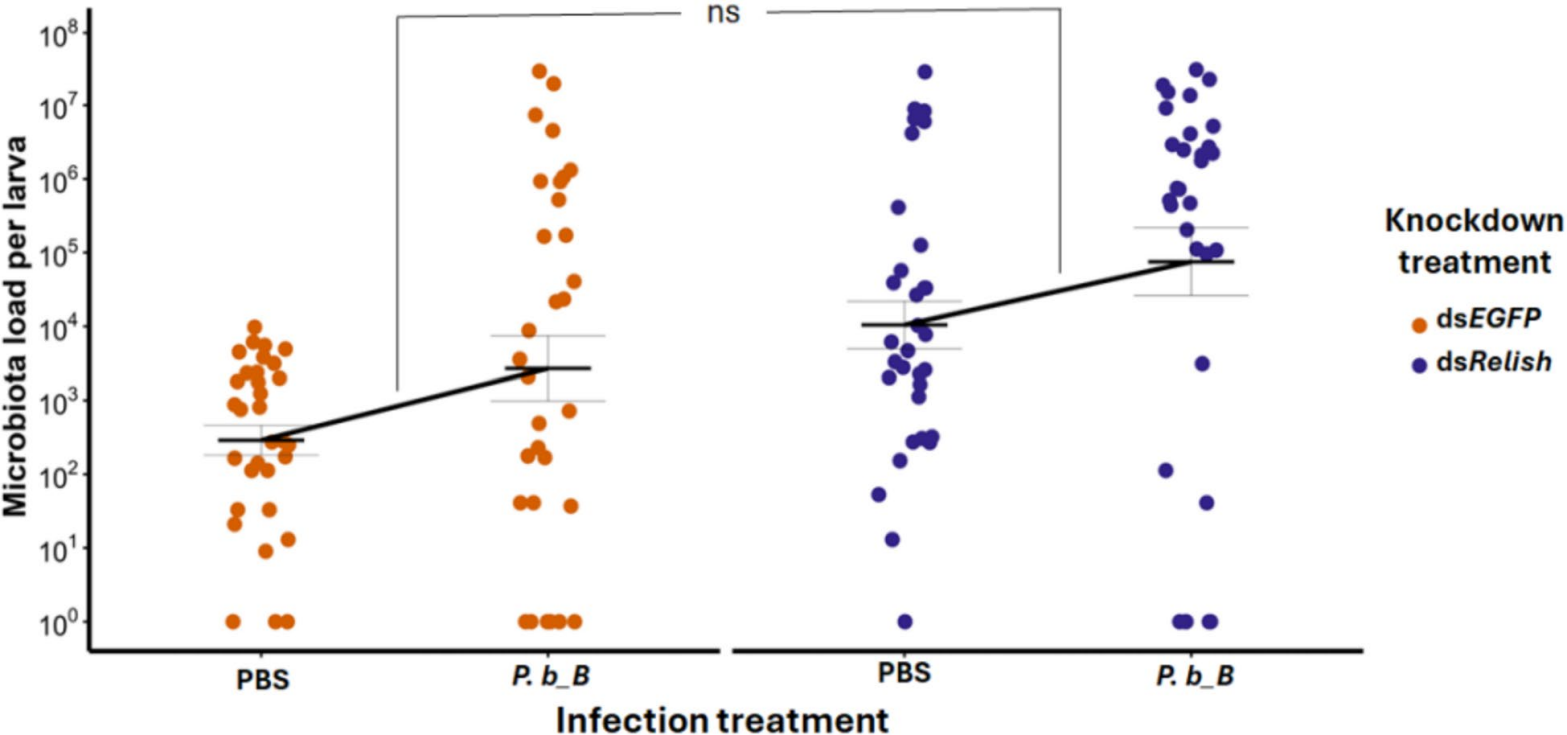
*TmRelish* knockdown and *Providencia burhodogranariea_B* infection effects on gut microbial abundance. Gut microbial load of all culturable bacteria in *Tenebrio molitor* larvae treated with ds*EGFP* (orange) and ds*TmRelish* (purple), following exposure to either *P. burhodogranariea_B* (*P. b_B*) or PBS at 24 h post-infection. Note that this measurement does not account for non-culturable bacteria. Means and standard errors are represented by the black lines, while the dots represent the biological replicates from individual guts (*n* = ∼16 per treatment, two independent experiments). We did not find support for our prediction (P1), as indicated by the statistically non-significant interaction between both predictors (X^2^_1, 127_ = 0.02, *p* = 0.896, scenario in Fig. 2e). However, our results suggest an indirect positive effect of infection on the gut microbiota as indicated by a significantly positive effect of infection (X^2^_1, 127_ = 4.87, *p* = 0.027, scenario in Fig. 2e). “ns” indicates no significant interaction between *TmRelish* knockdown and infection

Here we aimed to determine whether *P. b_B* infection indirectly affects gut microbiota load and to further test our first prediction (P1) that such an indirect interaction should be attenuated or absent in *TmRelish*-knockdown larvae. For this purpose, we first quantified culturable bacterial loads in individual guts from ds*EGFP-* and ds*TmRelish*-treated larvae following either PBS or *P. b_B* injection. Total bacterial load, including *P.b_B*, was estimated using non-selective TSA plates. To obtain microbiota load, we subtracted the antibiotic-resistant *P. b_B* count, measured on TSA supplemented with antibiotics, from the total bacterial load. In our statistical analysis, we did not find support for our prediction (P1) as there was no statistically significant interaction between both predictors, i.e., we found no indication that the infection-induced increase in gut microbiota load differs between *EGFP* controls and *TmRelish* knockdown (X^2^_1, 127_ = 0.02, *p* = 0.896, Fig. 6; Additional file 1: Fig. S3). Nevertheless, we found an indication that there is an indirect positive effect of *P. b_B* infection on the gut microbiota as gut microbiota load was higher in infected individuals (infection: X^2^_1, 127_ = 4.87, *p* = 0.027, scenario in Fig. 2e).

In addition, we found a statistically significant positive effect of *TmRelish* knockdown on gut microbiota load (ds*EGFP*/ds*TmRelish*: X^2^_1, 127_ = 20.30, *p* < 0.001, Fig. 6; Additional file 1: Fig. S3). This finding is consistent with the previously reported bipartite effect of Imd-dependent AMP expression on the gut microbiota (Fig. 3). Based on our linear model, we found no indication for differences in the magnitudes between the effects of infection and knockdown treatments. *TmRelish* knockdown increased the microbiota load by a factor of ∼36 (ds*TmRelish* vs. PBS across both diet treatments, 95% CI: 7.60, 172.44), while it was ~ 9.37 times higher for *P. b_B* infection (ds*EGFP* vs. *P. b_B* across both infection treatments, 95% CI: 1.28, 68.35). Due to the widely overlapping confidence intervals, we found no indication that microbiota load increased by different magnitudes following *TmRelish* knockdown and infection.

### The effect of gut microbiota on pathogen infections in tripartite interactions

**Fig. 7:**
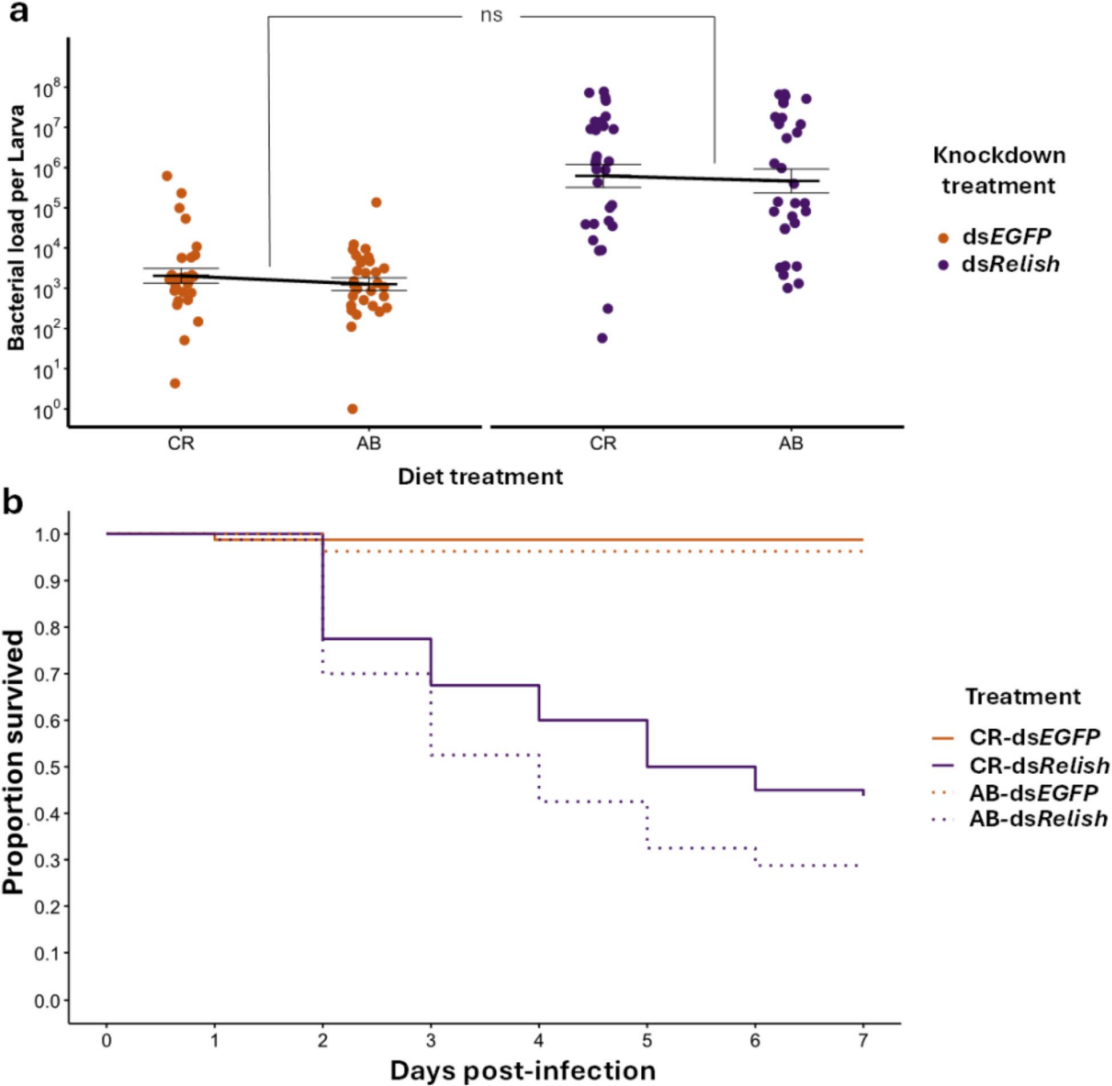
Effect of gut microbiota on *Providencia burhodogranariea_B* load and host survival in *TmRelish*-knockdown larvae. **a** *P. b_B* load of control-treated (CR) or antibiotic-treated (AB) larvae treated with ds*EGFP* (orange) and ds*TmRelish* (purple) at 24 h post-infection. Means and standard errors are represented by the black lines, while the dots represent the biological replicates from individual guts (*n* = 10 per treatment, three independent experiments). While we did not find a statistically significant interaction between both predictors (X^2^_1, 120_ = 0.031, *p* = 0.858, indicated in the figure by “ns”), *P. b_B* load was higher in *TmRelish*-knockdown larvae (ds*EGFP*/ds*TmRelish*: X^2^_1, 120_ = 61.883, *p* < 0.001). **b** Survival of CR or AB larvae injected with ds*EGFP* and ds*TmRelish* (*n* = ∼10 per treatment, eight independent experiments) following *P. burhodogranariea_B* (*P. b_B*) infection was monitored over a period of 7 days. While we did not find a statistically significant interaction between both predictors (X^2^_1, 32_ = 0.148, *p* = 0.699), gut microbiota and *TmRelish* knockdown had a statistically significant effect (CR/AB: X^2^_1, 32_ = 4.632, *p* < 0.01; ds*EGFP*/ds*TmRelish*: X.^2^_1, 32_ = 62.264, *p* < 0.001)

Here we aimed to assess potential indirect interactions between gut microbiota and *P. b_B* infection dynamics and to evaluate their consequences for host survival. In addition, we aimed to test our second prediction (P2) that the influence of gut microbiota on pathogen load should be absent or reduced in larvae with *TmRelish* knockdown. For this purpose, we used antibiotic-treated (AB) and control (CR) larvae following *TmRelish* knockdown during infection. When assessing *P. b_B* load at 24 h post-infection, we found no support for prediction P2, as there was no significant interaction between gut microbiota and knockdown treatments (X^2^_1, 120_ = 0.031, *p* = 0.858, Fig. 7a; Additional file 1: Fig. S4). We also found no indication for an indirect effect of gut microbiota on pathogen load as gut microbiota had no statistically significant effect on pathogen load (X^2^_1, 120_ = 0.772, *p* = 0.380, Fig. 7a, scenario in Additional file 1: Fig. S1a). Nevertheless, we recovered the previously observed bipartite effect of *TmRelish* knockdown on *P. b_B* load, with significantly higher loads in *TmRelish*-knockdown larvae (X^2^_1, 120_ = 61.883, *p* < 0.001, Figs. 4a and 7a).

In addition, we further tested the effects of gut microbiota and *TmRelish* knockdown on host mortality on day 7 post-infection. We found no support for our hypothesis that Imd-mediated AMPs mediates an indirect effect of gut microbiota on host survival during infection, as indicated by non-significant interaction between gut microbiota and knockdown treatments (X^2^_1, 32_ = 0.148, *p* = 0.699) (Fig. 7b). However, we observed higher mortality on day 7 in AB-treated larvae compared to CR-treated larvae (X^2^_1, 32_ = 4.632, *p* < 0.05, Fig. 7b), indicating an indirect positive effect of the gut microbiota on host survival during infection. Consistent with our bipartite interaction, we found higher mortality in ds*TmRelish*-treated larvae compared to ds*EGFP* larvae (X^2^_1, 32_ = 62.264, *p* < 0.001, Fig. 7b). These results suggest that both the gut microbiota and Imd-mediated AMP expression contribute independently to host survival. Therefore, the positive effect of gut microbiota on host survival seems to be mediated by other pathways. Of note, we confirmed that *P. b_B* remained viable in the AB-treated group, suggesting no toxicity of antibiotic, as demonstrated in a separate experiment (Additional file 1: Fig. S5).

### AMP expression in tripartite interactions

**Fig. 8:**
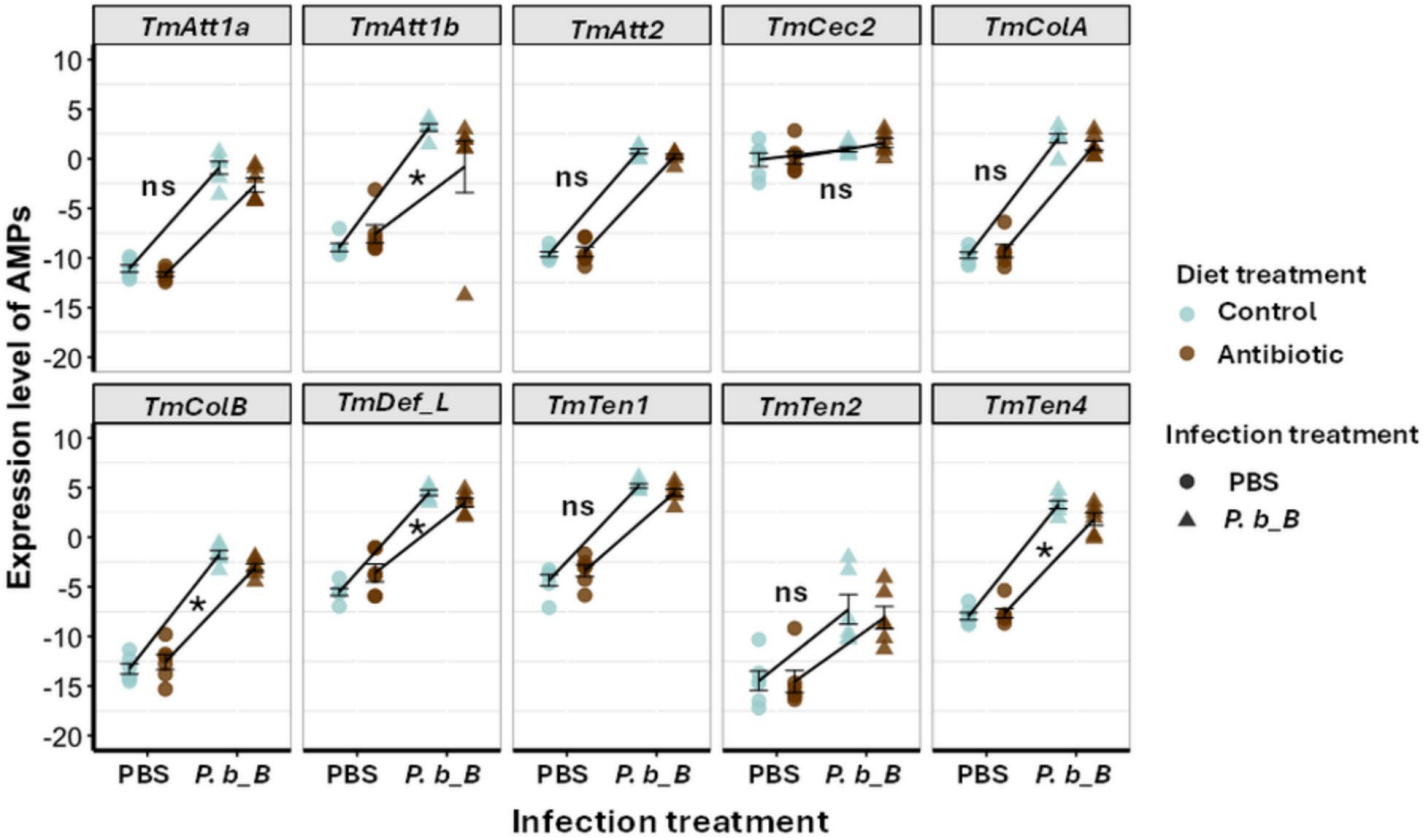
Regulation of antimicrobial peptide expression by gut microbiota in response to *Providencia burhodogranariea_B* infection. RT-qPCR analysis of AMP gene expression including *TmAtt1a*, *TmAtta1b*, *TmAtt2*, *TmTene1*, *TmTene2*, *TmTene4*, *TmColA*, *TmColB*, *TmCec2*, and *TmDef*-*L* in whole-body extracts of control (cyan) or antibiotic (brown) treated *Tenebrio molitor* larvae collected 24 h after infection with *P. burhodogranariea_B* (*P. b_B*, triangles). PBS-injected larvae (circles) were used as the mock control. Expression levels were normalized to *T. molitor 60S ribosomal protein L27a* (*TmL27a*) using the delta Ct method (mean Ct of *TmL27a* − mean Ct of AMP gene). Data points represent individual biological replicates, each with two technical replicates (*n* = 6 larvae per treatment), and error bars denote the mean ± SEM. Asterisks indicate significant interaction between gut microbiota and infection; ns, non-significant interaction

Our finding that the gut microbiota has no apparent effect on pathogen load at 24 h post-infection (Fig. 7a) suggests that improved host survival (Fig. 7b) is not mediated by a microbiota-induced increase in host resistance to infection. Nevertheless, to more directly investigate whether the gut microbiota contributes to host resistance mechanisms, we measured AMP transcript levels in CR- and AB-treated larvae following infection with *P. b_B* or PBS control treatment. For five AMPs, namely *TmAtt1a*, *TmTen1*, *TmTen2*, *TmColA*, and *TmCec2*, we found no statistically significant interaction between gut microbiota and infection (Fig. 8, Additional file 1: Table S2), indicating no evidence that the gut microbiota modulated their infection-induced upregulation. In contrast, for four AMPs, including *TmAtt1b*, *TmTen4*, *TmColB*, and *TmDef_L*, we detected a significant interaction between gut microbiota and infection, suggesting that the microbiota modulated their expression in response to infection. These findings suggest that the gut microbiota can modulate certain host resistance mechanisms. However, the microbiota-associated differences in AMP upregulation were limited to a subset of AMPs and relatively small compared to the overall magnitude of infection-induced expression (Fig. 8). This may explain why these differences had no apparent effect on bacterial load (Fig. 7a).

## Discussion

In this study, we confirmed that bipartite interactions between the *Tenebrio* immune system and either gut microbiota or *P. burhodogranariea_B* infection are mediated through Imd-dependent AMPs. In tripartite interactions, we found no indication that indirect interactions between gut microbiota and pathogens are mediated by Imd pathway. This suggests that other mechanisms, such as Imd-independent immune responses, host metabolic or within-host environmental factors (e.g., pH gradients, oxygen level), modulate indirect interactions. Our findings align with the emerging paradigm of tripartite interactions (host–pathogen-microbiota) in which variation in host immunity can influence gut microbiota dynamics and impose selective pressure on pathogens [1].

Our results, in line with observations in other insects, confirm an important role of the Imd pathway (assessed via the function of its downstream transcription factor, *TmRelish*) in regulating gut microbiota load and composition in *Tenebrio* larvae. The insect immune system, particularly Imd-mediated AMPs, has been implicated in shaping gut microbiota by selectively modulating bacterial populations, preserving homeostasis, and preventing dysbiosis [24, 44]. Consistent with *Drosophila* studies [22, 24], *TmRelish*-knockdown larvae exhibited an increase in gut microbial load and significant shifts in microbial composition. Here, we found that the genera *Bacillus* and *Pediococcus*, both Gram-positive bacteria with DAP-type peptidoglycan that activate the Imd pathway via peptidoglycan recognition proteins [45, 46], were significantly reduced in ds*TmRelish*-treated larvae compared to controls. This suggests that *TmRelish* plays a key role in regulating their proliferation (Additional file 1: Fig. S1b–d, c–e). Interestingly, some gut microbes, such as *L. plantarum* in *D. melanogaster* and *Lactobacillus apis* (strain W8172) in the honeybees, *Apis mellifera*, are known to induce host immune responses while remaining resistant to the host AMPs [21, 47]. Such immune modulation allows them to avoid being targeted while promoting the suppression of potential competitors. Similarly, in *T. molitor*, *Bacillus* and *Pediococcus* may activate the Imd pathways, which could limit the growth of less resistance competitors. Accordingly, in *TmRelish*-knockdown larvae, these genera might lose their competitive advantage, leading to the observed reduction in their abundance. *TmRelish* knockdown significantly increased larval mortality following *P. b_B* infection, coinciding with enhanced bacterial survival in ds*TmRelish*-treated larvae (Fig. 4). This susceptibility is likely associated with the suppression of *AMP* families, including *TmAttacin* (except *TmAtt2*), *TmTenecin*, and *TmColeoptericin*. The expression of *TmAtt2*, *TmDef_L*, and *TmCec2* was not significantly affected, suggesting that these *AMP*s might be regulated by alternative immune pathways independent of *TmRelish* (Fig. 5). While *TmCec2* is generally effective against Gram-negative bacteria such as *E. coli* [30, 48], its expression was not significantly induced in response to *P. b_B* infection. Of note, AMP expression in non-infected larvae remained largely unchanged (Fig. 5), underscoring the role of *TmRelish* in infection-specific immune modulation. These results align with previous studies showing that the Imd pathway recognizes pathogen-associated molecular patterns via peptidoglycan recognition proteins, triggering AMP production [49]. In *T. molitor* and other insects, Imd-mediated AMPs play a crucial role in defending against Gram-negative bacteria.

Despite the well-established role of Imd-mediated AMPs in shaping bipartite interactions, contrary to our initial hypothesis, our results do not support their involvement in mediating indirect interactions between gut microbiota and *P. b_B* infection (Figs. 6 and 7). While previous studies in tripartite contexts have highlighted the involvement of host immunity, such as the Toll pathway and Duox in mosquitoes [15, 19], we found no evidence that the Imd pathway serves a similar function in *T. molitor*. A possible explanation is that AMPs are also regulated by other pathways, which could, in turn, influence indirect interactions. In *TmRelish*-knockdown larvae, during infections AMPs were less strongly but still clearly upregulated (Fig. 5), suggesting that residual AMPs, potentially expressed through crosstalk with other immune pathways, might still have potential effects. Alternatively, because the transcriptional expression of *TmRelish* in knockdown larvae was not completely abolished (knockdown efficiency: 77.5%; Additional file 1: Fig. S4), the remaining *Tm*Relish protein may retain partial functionality sufficient to express AMPs.

That AMPs were still expressed despite *TmRelish* knockdown could be also explained by the immune system architecture of *T. molitor*, which is similar to that of other insects. In *Drosophila*, AMPs are typically expressed in response to Gram-negative and Gram-positive bacteria via the Imd and Toll pathways, respectively. However, studies in *Drosophila* and *Bombyx mori* suggest functional crosstalk between these pathways [50, 51]. For example, in *Drosophila*, heterodimers of the NF-κB transcription factors, dorsal-related immunity factor and *Relish*, have been shown to regulate AMP genes. Similarly, earlier studies in *T. molitor* have shown that *TmTen4* is induced by multiple ligands, including those activating both the Toll pathway (e.g., β−1,3-glucan and lysine-type peptidoglycan) and the Imd pathway (e.g., meso-DAP-type peptidoglycan), suggesting a broader immune response [36]. It should also be noted that AMPs alone may be insufficient to mediate indirect effects in *Tenebrio*. Earlier studies in *Drosophila* have revealed the crosstalk between ROS and AMPs in regulating the indirect interactions between gut microbiota and pathogens. ROS, produced by Duox in response to pathogen infection, can regulate the Imd pathway and potentially AMP activity, thereby altering gut microbiota load and composition [31]. Here, we focused only on the effects of Imd-mediated AMPs, rather than on the impact of other pathways like Duox-ROS. This may explain the lack of significant indirect interactions in our study. In another study, *Wolbachia*-infected *Drosophila* showed increased resistance to viral infections; although this effect can be explained by immune priming [20], pathogen blocking or modulation can also occur through non-immune mechanisms, such as microbial competition for host-derived resources (e.g., cholesterol and other lipids) or alteration of host cellular processes (e.g., vesicular trafficking) [52–54]. Another Imd-independent mechanism that could modulate the indirect interaction is host metabolism, which plays a crucial role in shaping infection outcome. For example, *Drosophila* infected with *Listeria monocytogenes* exhibit significant metabolic alterations, including shifts in lipid and carbohydrate metabolism, which contribute to immune function [55]. Together, these examples highlight that indirect interactions between microbiota and pathogens may be governed by a combination of metabolic-immune crosstalk and immune-independent physiological mechanisms. Alternatively, it is possible that indirect interactions do occur but were not detected due to experimental limitations. For instance, the route of infection or time-dependent dynamics may influence these interactions, and if they only manifest at specific time points, our sampling may have missed these effects.

Our investigations of tripartite interactions also demonstrate a positive effect, between microbes, where *P. b_B* infection promotes gut microbiota load (Fig. 6). This aligns with previous studies, such as fungal infection in *A. stephensi*, which altered immune responses in a way that promoted the gut microbiota growth [15]. Similarly, De Lorgeril et al. [16] observed a comparable pattern in the oyster *C. gigas*, where viral infection suppressed immune responses, enabling microbiota to flourish. In both cases, as in our study, the pathogen indirectly promoted microbiota growth, reinforcing the idea that immune suppression or dysregulation can create favorable conditions for both microbiota and pathogens to coexist temporarily, until host mortality disrupts this balance [56].

Our bacterial load data at 24 h post-infection showed no indication for an indirect effect of the gut microbiota on pathogen load (Fig. 7a). This suggests that the observed microbiota-dependent upregulation of four AMPs (Fig. 8) did not translate into lower *P. b_B* loads (Fig. 7a). Accordingly, we found no indication that the gut microbiota does modulate host resistance during infection. This could reflect that the observed AMP upregulation is insufficient to restrict *P. b_B* proliferation and that other mechanisms, such as competition for host resources or modulation of host cellular processes, may also contribute to protection. It is important to note that antibiotic treatment did not result in a complete depletion of the gut microbiota and additionally induced a significant shift in gut microbiota composition (i.e., larvae were not germ-free) (Additional file 2: B). This suggests that our ability to fully test prediction 2 was likely limited by the persistence of residual microbes. The higher mortality observed in AB-treated compared to CR-treated larvae (Fig. 7b) highlights a microbiota-mediated increase in host tolerance rather than resistance, a pattern also reported in other systems, including insects, where tolerance improved survival without reducing pathogen loads [57–60]. For example, in *A. mellifera*, the gut microbiota promotes viral tolerance by enhancing host survival during infection without reducing viral load [58]. Similarly, in *Drosophila*, *Wolbachia* enhance tolerance to flock house virus infection [20], and in *Culex pipiens*, it increases tolerance to *Plasmodium relictum*, the avian malaria parasite [61]. Accordingly, tripartite interactions not only shape infection outcomes but also influence the evolutionary trajectories of both the host and the pathogen [59, 60, 62], potentially driving pathogen virulence in either direction, either facilitating or constraining it [1].

## Conclusions

Gut microbiota dynamic within host–pathogen interaction can either facilitate or prevent infections. Our findings provide insight into the indirect interactions between gut microbiota and pathogens, mediated through Imd-dependent AMPs and Imd-independent mechanisms. In summary, our model confirms bipartite interactions between *Tenebrio* Imd-dependent AMPs and either gut microbiota or *P. b_B* infection, and bipartite interaction between the pathogen and the microbiota. However, in tripartite interactions, we found no evidence that indirect gut microbiota-pathogen interactions are mediated by Imd-dependent AMPs. Instead, it remains possible that other mechanisms, such as Imd-independent AMPs, host metabolism, microbiota-driven competition for resources, and host environment, might play a role and warrant further investigation. Host and microbiota-derived mechanism thus shape the interactions between gut microbiota and pathogens, maintaining microbial balance while defending against infections.

## Methods

### Tenebrio molitor rearing

Late instar larvae (18th to 20th instar, approximately 2.5–3 cm in length) were obtained from a commercial supplier (Reptile Food Handels-u. Zucht GmbH, Berlin, Germany) and maintained in cohorts of 500 larvae in the dark at 25 ± 3 °C and 60 ± 5% relative humidity. The larvae were provided with an ad libitum supply of wheat bran, serving as their primary food source. For hydration and supplementary nutrition, a fresh apple slice was added to each container every 48 h. At each feeding, the larvae were carefully checked, and newly pupated individuals were separated from the colony. Newly emerged adults were then transferred to separate containers for oviposition.

### Generation of antibiotic-treated larvae

*Tenebrio* larvae (8th to 9th instar, approximately 0.9–1 cm in length) were fed on a control diet (CR) consisting of 100 g of wheat bran, 10 g soy protein, 20 g soy flour, 171 g of a 5% yeast and wheat flour mixture, and 0.2 mL propionic acid in 200 mL of distilled water for 5 days to establish similar gut microbiota composition, whereas early instar larvae (3rd to 4th instar, approximately 0.5 cm in length) were fed an antibiotic-treated diet (AB) consisting of 100 g of wheat bran, 10 g soy protein, 20 g soy flour, 170 g of a 5% yeast and wheat flour mixture, 0.5 g of chloramphenicol, 0.5 g of sorbic acid, and 0.5 mL of propionic acid in 200 mL of distilled water for 15 days. Sorbic acid was included as a preservative to inhibit fungal growth in the moist diet. The last 2 days of feeding on the AB diet were limited to a diet with similar ingredients, except without chloramphenicol, to reduce the effect of the antibiotic. Fresh food was provided every 48 h to avoid desiccation.

### Bacterial culture

*P. burhodogranariea_B* (provided by Sophie Armitage) was retrieved from a 50% glycerol stock and streaked onto Luria–Bertani (LB) agar plates [63]. Single colonies were inoculated into 100 mL of LB broth and cultivated under aerobic conditions at 30 °C for 16 h. Following incubation, 50 mL of the overnight culture was centrifuged at 2880 × g for 10 min at 4 °C. The resulting cell pellet was washed twice with 40 mL of phosphate buffered saline (PBS) and resuspended in 5 mL of PBS. Optical density at 600 nm (OD600) was measured using spectrophotometer (Ultrospec 10, Amersham Biosciences) across 1:10, 1:100, and 1:1000 dilutions. Then, bacterial concentrations were calculated based on the linear equation for *P. burhodogranariea_B* (*y* = 9 × 10⁸ (*x*) − 1 × 10^7^), allowing the desired colony forming units per microliter (CFU/mL).

### DNA extraction and bacterial 16S sequencing analysis

DNA was extracted from homogenized larval gut tissue using DNeasy PowerSoil Pro Kit (Qiagen, Germany) with the following modifications. Larval guts were collected in 2-mL microcentrifuge tubes (Safe-Lock tubes, Eppendorf) containing 800 µL of CD1 solution buffer. After addition of two stainless steel beads (Ø 3 mm, Retsch), samples were homogenized using a tissue homogenizer (Mill MM400, Retsch) at 25 Hz for 10 min. The resulting homogenates were incubated overnight with 10 µL of proteinase K at 56 °C for 16 h in a ThermoMixer® C. The remaining steps were carried out according to the manufacturer’s protocol. DNA concentration was then measured via Qubit™ (Invitrogen) and subsequently diluted to 5 ng/µL.

The V2-V3 region of the 16S rRNA (16S ribosomal ribonucleic acid) encoding gene was amplified using 10 ng DNA (or negative control) as template, 1.25 µL of 10 µM primers 515 forward (515F) and 806 reverse (806R) (Additional file 1: Table S3), and 12.5 µL of Q5 High Fidelity Mastermix (New England Biolabs) in a 25 µL reaction under the following conditions: 98 °C for 30 s, 30 cycles of 98 °C, touch down 63–53 °C (− 1 K every second cycle) 30 s, 72 °C 10 s, and a final extension step 72 °C 120 s. The PCR amplicons were purified using CleanNGS CNGS-0050 (GC Biotech B.V.) and indexed with unique indexing primer combinations: 10 µL purified PCR1, 1.25 µL 10 µM indexing primer P5, 1.25 µL indexing primer P7, 12.5 µL Q5 High Fidelity Mastermix (New England Biolabs) with these reaction conditions: 98 °C 30 s; 8 cycles of 98 °C 10 s, 67 °C 30 s, 72 °C 30 s; final extension 72 °C 120 s as mentioned above. Libraries were sequenced using an Illumina MiSeq (Illumina) using sequencing kit v3 600 cycles at the Berlin Center for Genomics in Biodiversity Research (BeGenDiv).

The primer sequences were removed from the raw reads using cutadapt [64] and their quality checked with FastQC [65]. Accordingly, the raw reads were further trimmed at 200 bp for both forward and reverse reads with the “filterAndTrim” function of the package “dada2” [66]. They were then processed into amplicon sequence variants (ASVs) using Divisive Amplicon Denoising Algorithm 2 (DADA2) pipeline in R (“dada2” package). Taxonomy was assigned to the ASVs with the “assignTaxonomy” function of the “dada2” package, which uses a naive Bayesian classifier method of [67], and by querying against the Ribosomal Database Project (RDP) reference database [68]. Reads identified as mitochondria or chloroplasts, commonly observed in insect gut samples, were excluded from further analysis [69]. Bray–Curtis’s dissimilarity was then used to assess beta diversity, and principal coordinate analysis (PCoA) was performed to visualize these differences in community structure.

### cDNA synthesis and generation of double-stranded RNA

To synthesize PCR-based double-stranded RNA targeting *T. molitor* Relish (GenBank accession No. EEZ97717.1), a cDNA fragment corresponding to the target gene was produced using RevertAid™ Premium First Strand-cDNA-Synthese kit (Invitrogen), with total RNA extracted from the late instar larvae exhibiting high expression as template (Direct-zol RNA Miniprep Plus Kits, ZYMO Research, Europe GmbH). Gene-specific primers containing a T7 polymerase promotor sequence (TAATACGACTCACTATAGGG) at the 5′ end (Additional file 1: Table S3) were used to amplify the 851 bp PCR product from the cDNA template (KAPA2G Fast ReadyMix PCR Kit, KAPA Biosystems). PCR cycling profile was as follows: 95 °C for 2 min, followed by 30 cycles of denaturation at 95 °C for 20 s, annealing at 56 °C for 30 s, and extension at 72 °C for 5 min. Similarly, EGFP target (508 bp), cloned into the plasmid (pGEM T-easy-GFP, Promega), was amplified using gene-specific primers (Additional file 1: Table S3). Following checking of amplicon size via 2% agarose gel, the PCR products were purified using the PCR/DNA Clean-Up Kit (Roboklon). The purified amplicons served as templates for in vitro transcription, using the HighYield T7 RNA Synthesis Kit (Jena Bioscience) in accordance with the manufacturer’s instructions. Next, the synthesized dsRNA was washed, and the resulting pellet was resuspended in nuclease-free water and stored at − 20 °C for future applications.

### RT-qPCR

Total RNA was isolated by homogenizing larvae using TRIzol reagent following the manufacturer’s instructions (Direct-zol RNA Miniprep Plus Kits, ZYMO Research, Europe GmbH). RNA purity was validated via NanoDrop™ 2000/2000c Spectrophotometers (Thermo Scientific, Wilmington, DE, USA). The resulting mRNA was stored at − 80 °C until being used for real time qPCR. To set up the RT-qPCR for different genes, an amount of 20 ng/µL of the extracted mRNA was used using Power SYBR™ Green RNA-to-CT™ 1-Step Kit (Applied Biosystems™) with forward and reverse primers in a 10 µL reaction as previously described [30]. Briefly, forward and reverse primers were mixed with Power SYBR™ Green RT-PCR mix, RT-enzyme mix, and nuclease-free water to amplify the following gene expression: *TmRelish* (to assess knockdown efficiency) and AMP genes including *TmTenecin-1*, *TmTenecin-2*, *TmTenecin*-*4*, *TmAttacin-1a*, *TmAttacin-1b*, *TmAttacin-2*, *TmDefensin*-*like*, *TmColeoptericin-A*, *TmColeoptericin-B*, *TmCecropin*, and *T. molitor 60S ribosomal protein L27a* (*TmL27a*) (Additional file 1: Table S3).

To assess the knockdown efficiency of *TmRelish*, conventional larvae were injected with 1000 ± 100 ng of dsRNA (1000 μg/μL in 1 μL) of both ds*TmRelish* and ds*EGFP* (negative control). Total RNA was extracted from a pool of three larvae per day (*n* = 3, three experimental replicates) at indicated time points of days 1, 3, 5, and 7 post-injection and in individual larvae at 3 days post-injection. RT-qPCR was performed as described. The mean Ct value of the gene of interest (GOI) was normalized using the mean Ct value of housekeeping gene by calculating delta Ct (mean Ct of housekeeping gene − mean Ct of GOI), where the housekeeping gene is *T. molitor 60S ribosomal protein L27a* (*TmL27a*) [70].

### Experimental procedures

All experimental individuals fed on either CR or AB were 9th to 10th instar larvae (approximately 1–1.5 cm in length) [71].

Given that diet shapes gut microbiota, to determine if chloramphenicol in the diet affects gut microbiota composition, individual guts of CR- and AB-treated larvae (*n* = 12 per treatment) were dissected and bead-grounded for DNA extraction. Differences between treatments were assessed using both culture-dependent and culture-independent approaches. Detailed procedures are provided in the supporting information (Additional file 1: Fig. S6 and Additional file 2: B).

#### Bipartite interactions

To assess the impact of *TmRelish* knockdown on the proliferation of *T. molitor* gut microbiota and composition, an RNAi knockdown construct targeted against *TmRelish* was expressed; accordingly, conventional larvae were treated with double-stranded RNA (dsRNA) of *Enhanced Green Fluorescent Protein* (ds*EGFP*) and ds*TmRelish*. We recapitulated our previous finding on knockdown efficiency; result was confirmed via qRT-PCR [30] (Additional file 1: Fig. S7 and Additional file 2: C). Each larva subjected to ds*TmRelish* or ds*EGFP* (*n* = ∼12 per treatment, six independent experiments) was dissected at 3 days post-exposure. Individuals were homogenized in 200 µL PBS using two sterile glass beads at 30 Hz for 20 s using a tissue homogenizer (Mill MM400, Retsch). Homogenates were serially diluted (1:10 to 1:10^5^) and plated onto the trypticase soy agar (TSA) plates [72].

To examine if *P. burhodogranariea_B* can evade the impaired immune system of *T. molitor* or is eliminated by it, larvae treated with ds*EGFP*-*P. b_B* and ds*TmRelish*-*P. b_B* (*n* = ∼10 per treatment per day, two independent experiments) were homogenized in 250 μL of LB broth at different time points (0-day, 1-day, 2-day, 3-day, 4-day, 5-day, 6-day, and 7-day post-infections). Homogenization was performed as described above, followed by centrifugation at 420 × g for 1 min at 4 °C. Homogenates from each individual were added into 180 μL of LB broth and then serially diluted (1:10 to 1:10^5^). *P. burhodogranariea_B* colonies were cultured on LB medium containing 16 mg/mL of nalidixic acid and 32 mg/mL of rifampicin for 16 h at 30 °C. Five drops (5 μL) per larvae were counted and averaged as replicates. In addition, we injected PBS in ds*EGFP* and ds*TmRelish* larvae as a negative control: no colonies were recovered from these larvae (*n* = 8).

To investigate whether *P. b_B* infection, *TmRelish* knockdown, and their interaction impact the host survival and *Relish*-dependent AMP expression, conventional larvae were first injected with ds*EGFP* or ds*TmRelish*. Three days post-dsRNA exposure, larvae were injected with 2 µL of PBS (control) or with *P. burhodogranariea_B* (*P. b_B*) at a concentration of 5 × 10^7^ colony forming units (CFU/mL), corresponding to a dose of approximately 10^5^ CFU per insect. The bacterial dose was selected as the highest dose that did not cause mortality (Additional file 1: Fig. S8).

For host survival analysis, each group of insects, including control (*P. b_B* without dsRNA injection, CR-*P. b_B*), ds*EGFP*-*P. b_B*, and ds*TmRelish*-*P. b_B* (*n* = ∼30 per treatment, three independent experiments), was monitored daily for 1 week. Additionally, we assessed *P. b_B* colonization in RNAi-treated larvae. For measuring the transcript level of targeted mRNA, i.e., *TmRelish*-mediated AMP expression, six larvae as a biological replicate were collected from larvae treated with ds*EGFP* and ds*TmRelish* followed infection with either PBS or *P. burhodogranariea_B* (ds*EGFP*-PBS, ds*TmRelish*-PBS, ds*EGFP*-*P. b_B*, ds*TmRelish*-*P. b_B*).

#### Tripartite interactions

Next, to investigate whether pathogen infection affects the gut microbiota and further assess whether this effect differs in *TmRelish*-knockdown individuals (Fig. 1a), conventional larvae treated with ds*EGFP* and ds*TmRelish* followed infection with either PBS or *P. burhodogranariea_B* (ds*EGFP*-PBS, ds*TmRelish*-PBS, ds*EGFP*-*P. b_B*, ds*TmRelish*-*P. b_B*) were dissected at 24 h post-infection and plated onto either TSA or TSA medium containing 16 mg/mL of nalidixic acid and 32 mg/mL of rifampicin. After 16 h of incubation at 37 °C, CFUs were counted from five (5 μL) drops per sample. Each individual served as a biological replicate (*n* = ∼16 per treatment, two independent experiments).

To test knockdown of *TmRelish* effects on bacterial load and host survival in the presence of either a control or disrupted gut microbiota (Fig. 1b), CR- and AB-treated larvae were injected with ds*EGFP* or ds*TmRelish* followed infection with *P. b_B*.  Each group of insects, including CR-ds*EGFP-P. b_B, CR-dsTmRelish-P. b_B, ABdsEGFP-P. b_B, and AB-dsTmRelish-P. b_B* was used to assess bacterial load at 24 h post-infection (n = 10 per treatment, three independent experiments) and monitored daily for 1 week in a survival assay (n ≈ 10 per treatment, eight independent experiments).

Additionally, we used RT-qPCR to investigate the effects of imbalanced gut microbiota on AMP expression. For this, we injected CR- and AB-treated larvae with PBS or with *P. b_B*. Two independent experimental replicates were conducted. In each experimental replicate and for each treatment, three larvae were injected, giving a total of 24 larvae across the four groups CR-PBS, AB-PBS, CR-*P. b_B*, and AB-*P. b_B*.

Of note, larvae were not surface sterilized prior to homogenization, in line with the recommendations by Hammer et al., suggesting that surface sterilization should be avoided in insect microbiota research, as it may inadvertently alter the composition of internal bacterial communities [73].

### Statistical analysis

All data were analyzed using R statistical software (v 4.4.1; R core team 2024) [74]. We fitted generalized linear mixed models (GLMMs), implemented in the “glmmTMB” package (version 1.1.9) [75], for the knockdown efficiency analysis, AMP expression analysis, survival assays, and gut microbiota load. When necessary, to better understand the effects of significant interactions, we performed pairwise post hoc tests using “emmeans” package (package version 1.10.4) [76]. Assessment of model assumptions were evaluated using the DHARMa package (version 0.4.6) [77].

In bipartite interaction between host and gut microbiota, for analysis of knockdown efficiency of gene expression, we evaluated the effects of knockdown treatment on delta Ct values. Here, delta Ct was defined as the mean Ct of housekeeping gene minus mean Ct of gene of interest, with delta Ct serving as response variable and replicates as a random effect. For gut microbial load, we fitted a GLMM with a negative binomial distribution, to assess the effect of *TmRelish* knockdown on gut microbial load. The response variable was gut microbial load (CFUs), with knockdown treatments (ds*EGFP* or ds*TmRelish*) as the fixed effect and experimenter and replicate as random effects. The amplicon sequence variant (ASV) abundance data from the 16S sequencing of the larval bacterial gut community generated by the DADA2 pipeline was imported and analyzed with the “phyloseq” package [78]. Reads identified as mitochondria, chloroplasts, or eukaryote plant material, commonly observed in insect gut samples, were excluded from further analysis [69]. Briefly, ASVs were agglomerated at the genus and order levels, and the data from the two experiments were subsetted and analyzed separately. Operational taxonomic units (OTUs) counts for each sample were normalized to the total number of OTUs per sample, and a Bray–Curtis dissimilarities matrix was built as an index of beta diversity. A PERMANOVA was performed with the “adonis” function of the “vegan” package with an alpha level of 0.05 as a threshold to determine significance [79]. The phyloseq data was then converted to a DESeq2 dataset, and the geometric mean, size factors, and dispersions of each OTU were estimated before performing a likelihood ratio test (LRT) to identify differentially abundant OTUs (alpha = 0.05) between treatments using “DESeq2” package [80].

In bipartite interaction between host and pathogen, for survivorship data we initially aimed to use a Cox proportional hazard model. However, preliminary analyses indicated a clear violation of the proportionality assumption. Therefore, we chose to use a GLMM, with mortality at day 7, defined as a binary outcome (number of dead or alive) as response variable, knockdown treatment (ds*EGFP* or ds*TmRelish*) as fixed effect, and date as a random effect to account for variability across days. To test the effects of knockdown treatment and time on log-transformed bacterial load as the response variable, we fitted a linear model, with knockdown treatment and time serving as fixed effects and replicate as a random effect. For AMP expression analysis, we fitted a GLMM to assess the effects knockdown treatment, infection, and their interaction as fixed effects on delta Ct values.

In tripartite interaction context, to evaluate the effects of knockdown treatment, infection, and their interaction on log-transformed gut microbiota load, we fitted a linear mixed model, with knockdown treatment and injection serving as fixed effects, gut microbiota load as response variable, and replicate as a random effect. We analyzed the data from bacterial load, survival, and AMP expression similar to bipartite interaction experiments. To evaluate the effects of knockdown treatment, gut microbiota, and their interaction on bacterial load, we fitted a linear mixed model, with log-transformed bacterial load as response variable, knockdown treatment and gut microbiota serving as fixed effects, and replicate as a random effect. For survivorship data, we used a GLMM, with mortality at day 7 as response variable, knockdown treatment (ds*EGFP* or ds*TmRelish*) and effects of gut microbiota (CR or AB treatments) as fixed effect, and date as a random effect. For AMP expression analysis, we fitted a GLMM to assess the effects of gut microbiota, infection, and their interaction as fixed effects on delta Ct values. 

## Supplementary Information

Additional file 1: Figures S1–S8; Tables S1–S3. Fig. S1 Conceptual predictions for indirect effects of gut microbiota load on *Providencia burhodogranariea_B* infection. Fig. S2 Microbiota composition and differential abundance in *Tenebrio molitor* larvae treated with ds*EGFP* and ds*TmRelish*. Fig. S3 *Relish* knockdown and *Providencia burhodogranariea_B* infection effect on gut microbiota. Fig. S4 *Relish* knockdown and gut microbiota effect on *Providencia burhodogranariea_B* load. Fig. S5 Viability of *P. burhodogranariea_B* in antibiotic-treated *Tenebrio molitor* larvae. Fig. S6 Microbiota composition and differential abundance in control and antibiotic-treated *Tenebrio molitor* larvae. Fig. S7 Knockdown efficiency of *TmRelish* in RNAi-treated *Tenebrio molitor* larvae. Fig. S8 Survival of *Tenebrio molitor* larvae upon infection with different doses of *P. burhodogranariea_B*. Table S1 Generalized linear mixed model results for the effects of *TmRelish* knockdown and infection on AMP expression. Table S2 Generalized linear mixed model results for the effects of gut microbiota and infection on AMP expression. Table S3 Primer sequences used in this study.

Additional file 2: Detailed results. A—Effect of *TmRelish* knockdown on gut composition in *Tenebrio* larvae. B—Effect of chloramphenicol on gut microbiota diversity in *Tenebrio* larvae. C—*Tenebrio molitor Relish* knockdown efficiency.

## Data Availability

The raw data set and R scripts used in all analyses are available from the corresponding author upon request.

## Abbreviations

AB: Antibiotic-treated larvae
AMPs: Antimicrobial peptides
CFU: Colony forming units
CR: Control-treated larvae
Duox: Dual oxidase
*EGFP*: *Enhanced Green Fluorescent Protein*
GLMMs: Generalized linear mixed models
Imd: Immune deficiency
OTUs: Operational taxonomic units
*P. b_B*: *Providencia burhodogranariea_B*
PBS: Phosphate buffered saline
*Tm*: *Tenebrio molitor*
*TmAtt1a*: *TmAttacin-1a*
*TmAtt1b*: *TmAttacin-1b*
*TmAtt2*: *TmAttacin-2*
*TmCec2*: *TmCecropin*
*TmColA*: *TmColeoptericin-A*
*TmColB*: *TmColeoptericin-B*
*TmDefL*: *TmDefensin-like*
*TmL27a*: *Tenebrio molitor 60S ribosomal protein L27a*
*TmTen1*: *TmTenecin-1*
*TmTen2*: *TmTenecin-2*
*TmTen4*: *TmTenecin-4*
TSA: Trypticase soy agar
