# Supplementary material for: Immune-mediated indirect interaction between gut microbiota and bacterial pathogens
Source: BMC Biol. 2025 Sep 16;23:278. doi: 10.1186/s12915-025-02399-1 (PMC12442303; doi:10.1186/s12915-025-02399-1)
Supplement: Supplementary file 1 — Additional file 1: Figures S1–S8; Tables S1–S3. Fig. S1 Conceptual predictions for indirect effects of gut microbiota load on Providencia burhodogranariea_B infection. Fig. S2 Microbiota composition and differential abundance in Tenebrio molitor larvae treated with dsEGFP and dsTmRelish. Fig. S3 Relish knockdown and Providencia burhodogranariea_B infection effect on gut microbiota. Fig. S4 Relish knockdown and gut microbiota effect on Providencia burhodogranariea_B load. Fig. S5 Viability of P. burhodogranariea_B in antibiotic-treated Tenebrio molitor larvae. Fig. S6 Microbiota composition and differential abundance in control and antibiotic-treated Tenebrio molitor larvae. Fig. S7 Knockdown efficiency of TmRelish in RNAi-treated Tenebrio molitor larvae. Fig. S8 Survival of Tenebrio molitor larvae upon infection with different doses of P. burhodogranariea_B. Table S1 Generalized linear mixed model results for the effects of TmRelish knockdown and infection on AMP expression. Table S2 Generalized linear mixed model results for the effects of gut microbiota and infection on AMP expression. Table S3 Primer sequences used in this study. [file 12915_2025_2399_MOESM1_ESM.docx]

**Additional file 1: Figures S1-S8; Tables S1-S3**


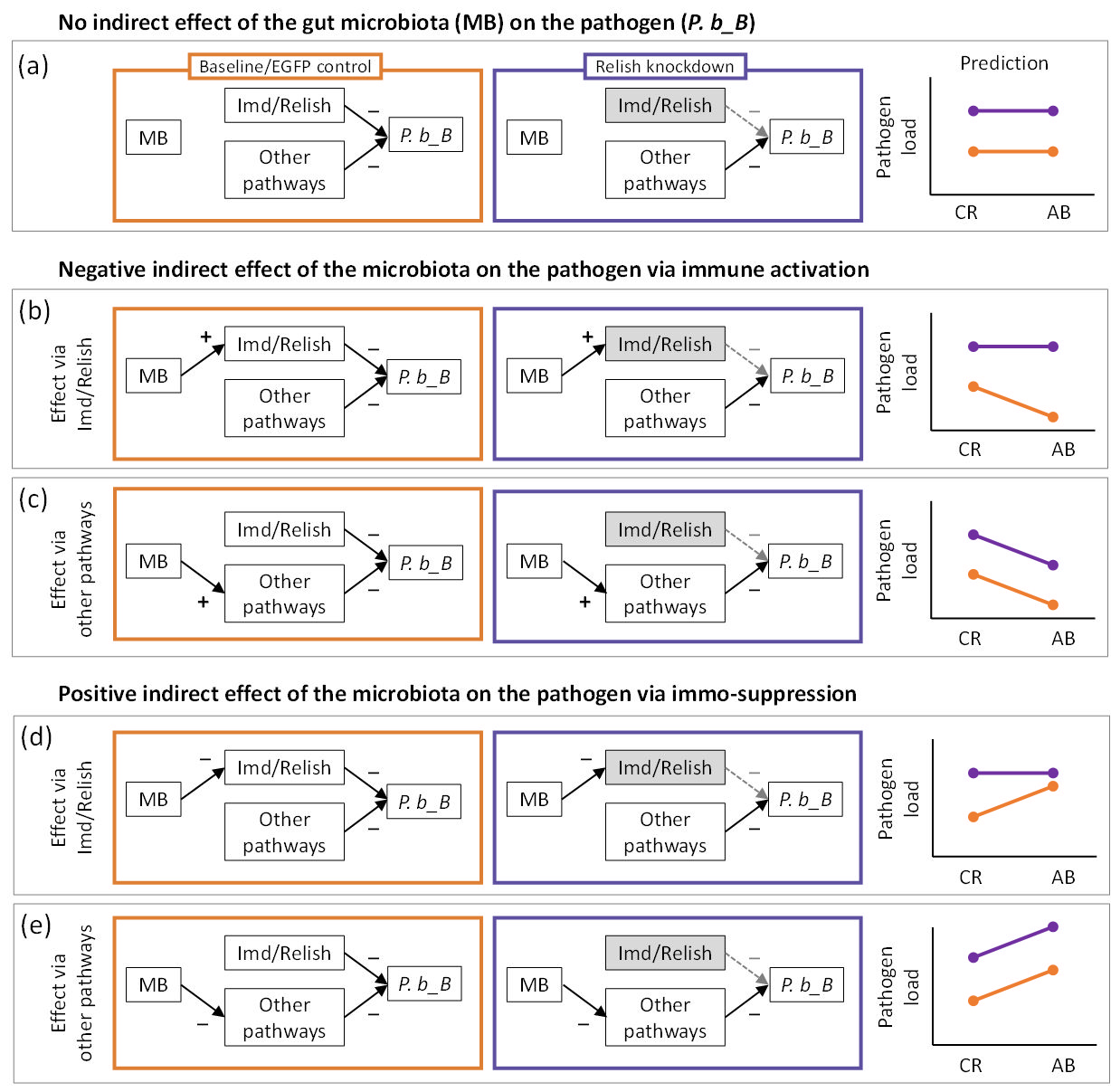


**Additional file 1: Figure S1.** **Conceptual predictions for indirect effects of gut microbiota load on *Providencia burhodogranariea_B* (*P. b_B*)** **infection.** In all depicted scenarios we assumed that pathogen proliferation is affected by Imd-dependent antimicrobial peptides (AMPs) (via *Relish*) and other immune pathways, such that immune activation exerts a negative effect on pathogen load. Orange boxes and arrows represent baseline (ds*EGFP* control) conditions, whereas purple indicates *Relish* knockdown conditions, in which Imd pathway is assumed to be fully silenced. (**a**) Baseline scenario in which the gut microbiota exerts no indirect effect on the pathogen. (**b**, **c**) Scenarios with a negative indirect effect (apparent competition) of the microbiota on the pathogen through immune activation. (**d**, **e**) Scenarios with a positive indirect effect (apparent mutualism) of the microbiota on the pathogen via immunosuppression. (**b**, **d**) In these scenarios, the indirect effect is mediated by Imd-dependent AMP expression. (**c**, **e**) In contrast, the indirect effect is mediated by other immune pathways independent of Imd-dependent AMP expression.


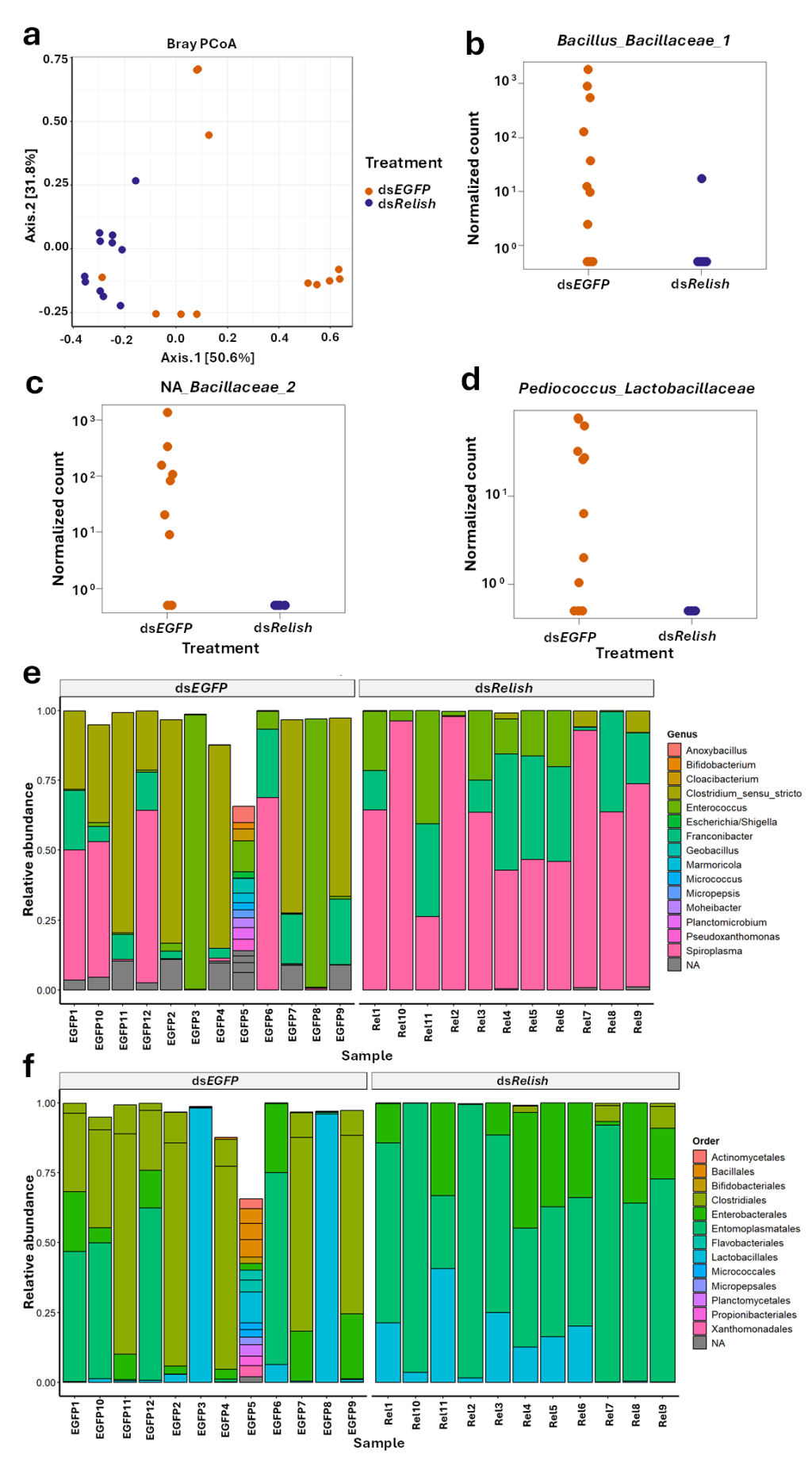


**Additional file 1: Figure S2. Microbiota composition, and differential abundance in *Tenebrio molitor* larvae treated with ds*EGFP* and ds*TmRelish*.** (**a**) Principal coordinates analysis (PcoA) plot visualizing Bray-Curtis dissimilarity of the composition of the bacterial gut microbiota between ds*EGFP* (orange) and ds*TmRelish* (purple) *T. molitor* larvae. Each point represents the gut microbiota composition of an individual larva, with colour indicating the treatment group. The axis labels indicate the percentage of variation captured by each dimension. A PERMANOVA with 999 permutations showed that the treatment significantly separates the samples. (**b**) ASV counts of an OTU member of the *Bacillus* genus (family *Bacillaceae*_1), (**c**) ASV counts of an OTU belonging to an unidentified genus (family *Bacillaceae*_2), and (**d**) ASV counts of an OTU member of the *Pediococcus* genus (family *Lactobacillaceae*) illustrated between ds*EGFP* (orange) and ds*TmRelish* (purple) larvae. Each point represents a single larva, with colours indicating the treatment. (**e**) Relative abundance of genera and (**f**) orders for the top 20 taxa detected by 16S rRNA gene sequencing in *T. molitor* larvae treated with dsRNA, visualized by bar plots. Each bar represents an individual sample, with coloured box indicating different taxa. The hight of each box represents to the relative abundance of that taxon within the samples. Grey boxes indicate OTUs for which no taxonomy could be assigned.


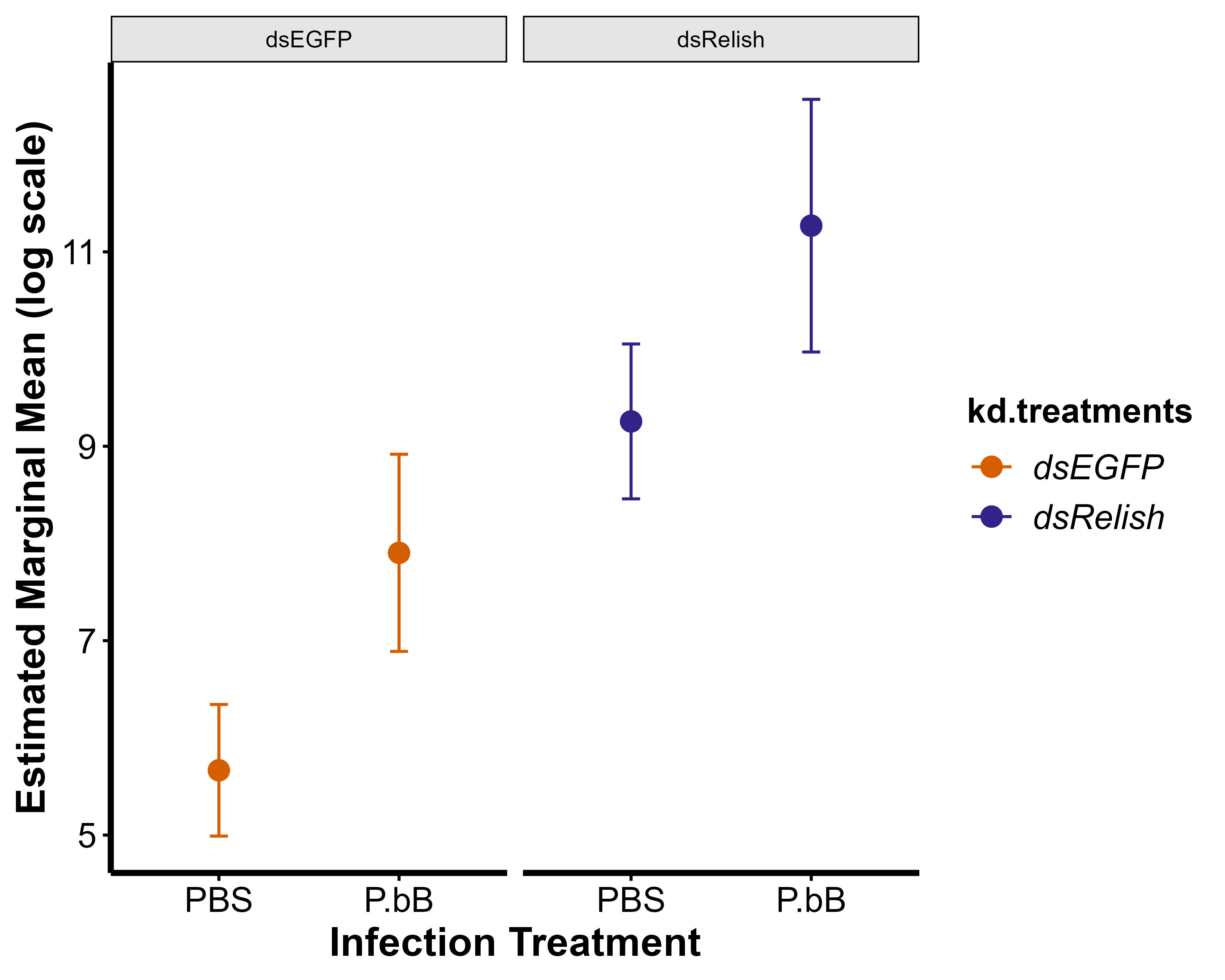


**Additional file 1: Figure S3. *Relish* knockdown and *Providencia burhodogranariea_B* infection effect on gut microbiota.** Estimated marginal means with 95% confidence intervals of gut microbial load in *Tenebrio molitor* larvae treated with ds*EGFP* (orange) and ds*TmRelish* (purple), following exposure to either *P. burhodogranariea_B* or PBS.


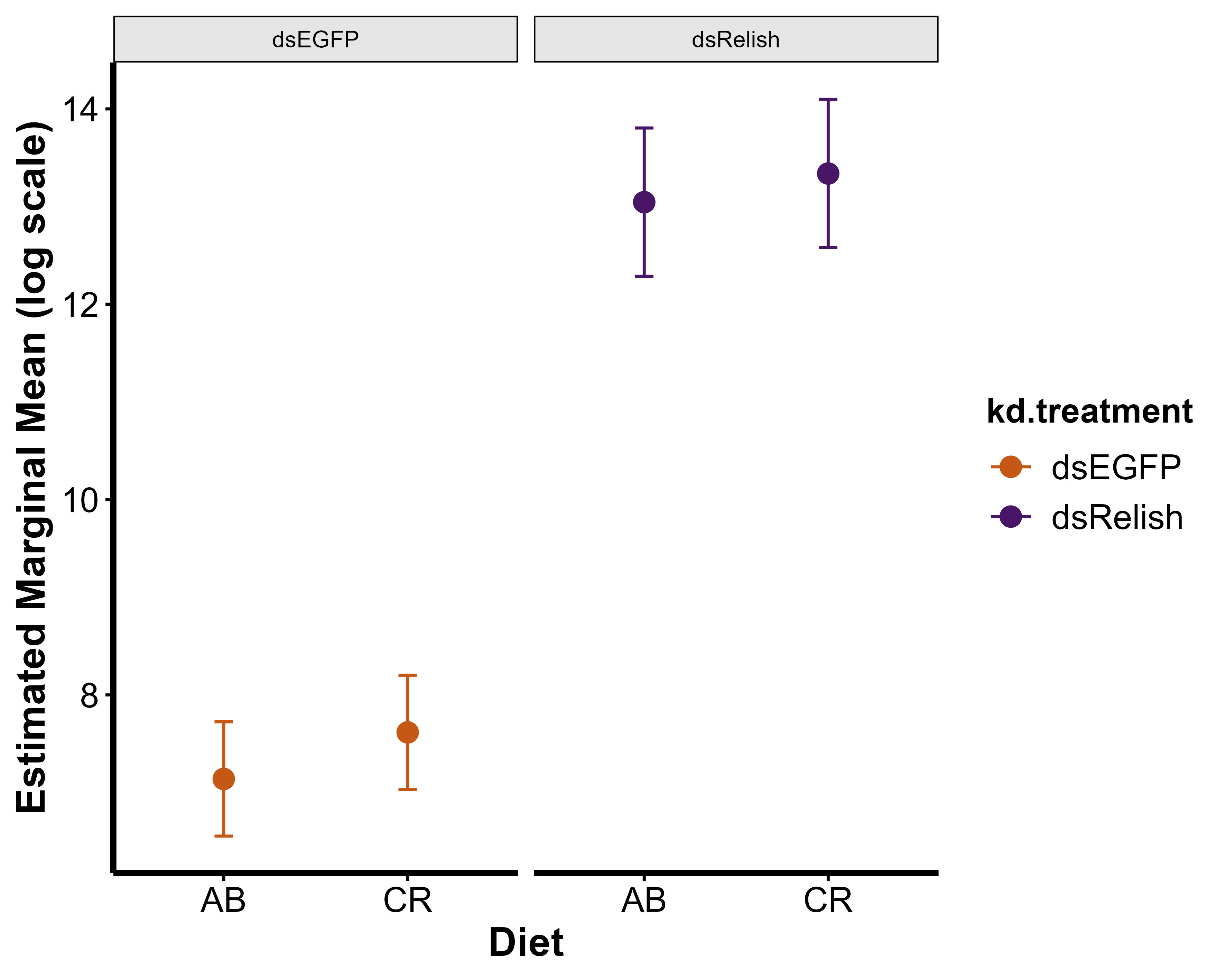


**Additional file 1: Figure S4. *Relish* knockdown and gut microbiota effect on *Providencia burhodogranariea_B* load.** Estimated marginal means with 95% confidence intervals of *Providencia burhodogranariea_B* load in antibiotic-treated (AB) or control-treated (CR) *Tenebrio molitor* larvae treated with ds*EGFP* (orange) and ds*TmRelish* (purple).


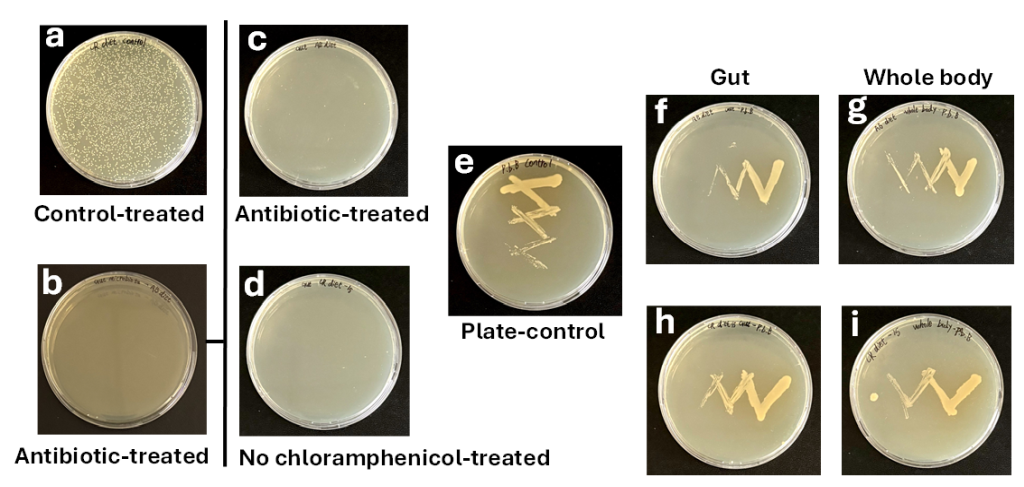


**Additional file 1: Figure S5. Viability of *P. burhodogranariea_B* (*P. b_B*) in antibiotic-treated *Tenebrio molitor* larvae.** Representative TSA plates exhibit the presence of viable *P. b_B* after feeding with different dietary treatments. (**a**) Guts of larvae fed a control diet (CR) and (**b**) guts of larvae fed an antibiotic-treated diet (AB) were dissected and plated on day 13 post-feeding. (**c**) Guts of AB-treated larvae and (**d**) guts of larvae fed a diet with the same ingredients but without chloramphenicol (No-chloramphenicol-treated) were plated on day 15 post-feeding. (**e**) *P. b_B* was streaked directly onto the plate as a positive control. For assessment of *P. b_B* presence, (**f**) dissected guts and (**g**) whole bodies of AB-treated larvae, and (**h**) dissected guts and (**i**) whole bodies of No-chloramphenicol-treated larvae were smeared onto plates alongside *P. b_B* streaks. All plates were incubated at 30 °C for 16 hours.


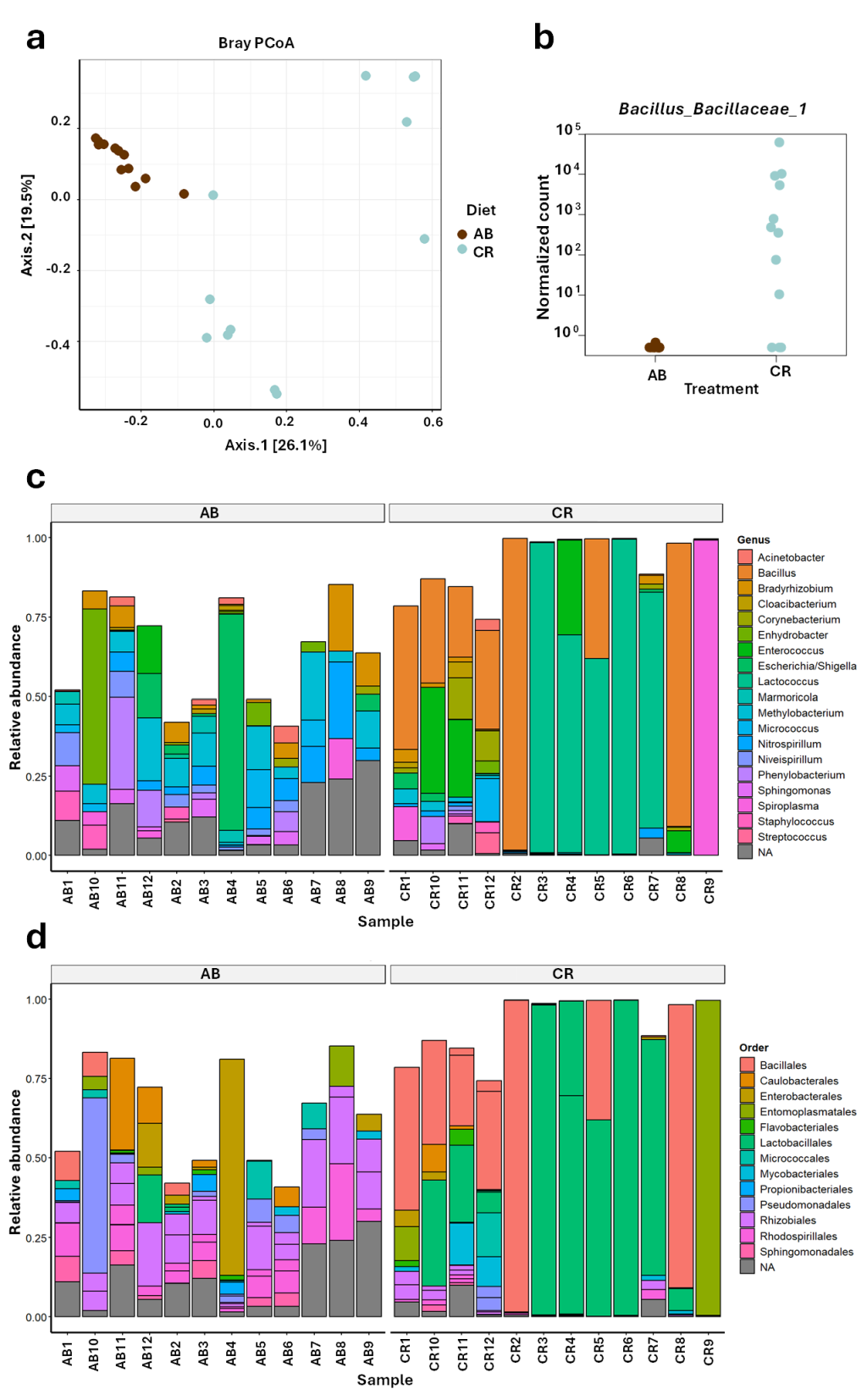


**Additional file 1: Figure S6.** **Microbiota composition and differential abundance in control and antibiotic-treated *Tenebrio molitor* larvae.** (**a**) Principal coordinates analysis (PcoA) plot visualizing Bray-Curtis dissimilarity in the composition of the bacterial gut microbiota between control (cyan) or antibiotic-treated (brown) *Tenebrio molitor* larvae (9^th^ to 10^th^ instar). Each point represents the Bray-Curtis index of an individual larva. The axis labels indicate the percentage of variation captured by each dimension. A PERMANOVA with 999 permutations showed that the treatment significantly separates the samples. (**b**) ASV counts of an OTU of the *Bacillus* genus (family *Bacillaceae*) whose abundance is significantly explained by the treatment (CR or AB). Each point represents the ASV count in a single *T. molitor* larva of the control (CR, cyan) and antibiotic-treated (AB, brown) groups. (**c**) Relative abundance of genera (**d**) and orders for the top 20 taxa detected by 16S rRNA gene sequencing in *T. molitor* larvae treated with different diets, visualized by bar plots. Each bar represents an individual sample, with coloured box indicating different taxa. The hight of each box represents the relative abundance of that taxon within the samples. Grey boxes indicate OTUs for which no taxonomy could be assigned.


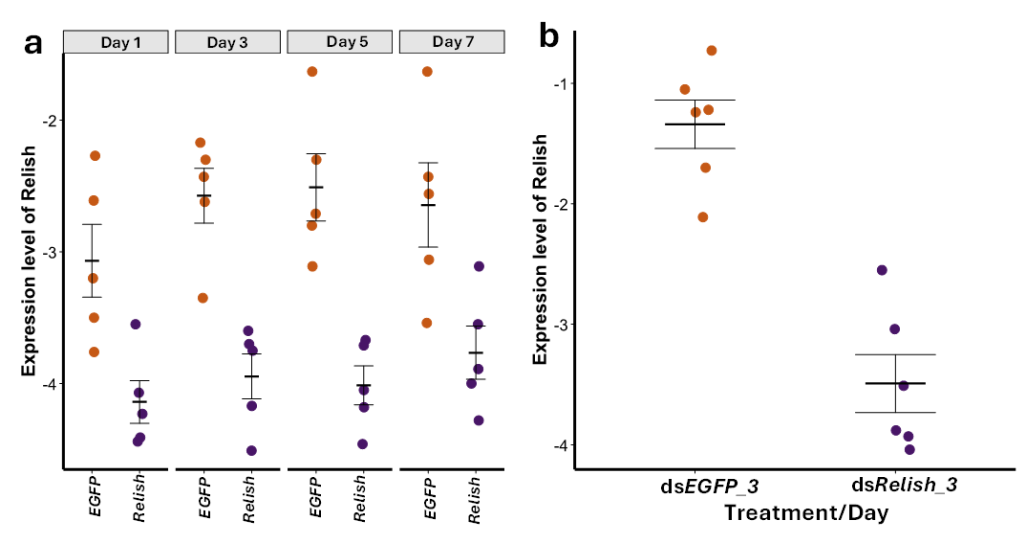


**Additional file 1: Figure S7. Knockdown efficiency of *TmRelish* in RNAi-treated *Tenebrio molitor* larvae.** (**a**) Quantitative measurement of pooled *Tenebrio molitor* *Relish* (*TmRelish*) mRNA levels in ds*TmRelish*-injected larvae (purple) determined by RT-qPCR. One microgram of dsRNA targeting *TmRelish* at the concentration of 1000 ng/μL in 1 μL were injected to 9^th^ to 10^th^ instar larvae. Total RNA was isolated on the 1^st^, 3^rd^, 5^th^, and 7^th^ day following treatments. Each data point represents a pool of three beetles. (**b**) Individual level knockdown efficiency of *TmRelish* on the third day post-exposure. Note, in contrast to (a) here samples were no pooled and each data point represent a single individual. ds*EGFP*/3 and ds*Relish*/3 indicate the treatment group (ds*EGFP* or ds*Relish*) and the sampling time point (day 3 post-exposure). The mRNA quantity of *TmRelish* was measured in relation to *T. molitor 60S ribosomal protein L27a* (*TmL27a*) as an internal control. EGFP RNAi was used as a negative control (orange). Evaluation of *TmRelish* mRNA expression levels after RNAi injection, compared to those in ds*EGFP*-treated, demonstrated a knockdown efficiency of 77.5%. The Ct values of the gene of interest were normalized to the Ct values of the reference gene.


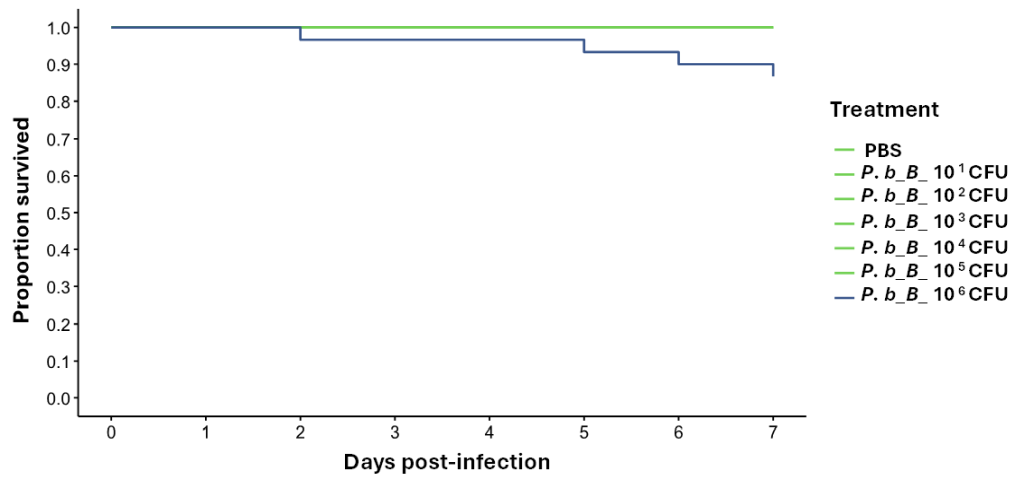


**Additional file 1: Figure S8. Survival of *Tenebrio molitor* larvae upon infection with different doses of *P. burhodogranariea_B*.** Conventional *T. molitor* (9^th^ to 10^th^ instar) larvae were infected with *P. burhodogranariea_B* (*P. b_B*) at doses of 10^1^, 10^2^, 10^3^, 10^4^, 10^5^(green), and 10^6^ (blue) CFU/insect (n=30 per treatment) and their survival was monitored over the course of seven days. PBS-injected larvae were used as control.

**Additional file 1: Table S1. Generalized linear mixed model results for the effects of *TmRelish* knockdown and infection on AMP expression.** Results of generalized linear mixed models testing the effects of *TmRelish* knockdown, infection and their interaction on AMP expression, separately for each AMP.

| Gene name | Predictor | estimate | SE | Z value | *p* value |
| --- | --- | --- | --- | --- | --- |
| *TmAtt1a* | **kd. treatment** | -7.587 | 0.774 | -9.806 | **< 0.001** |
|  | **infection** | -10.988 | 0.774 | -14.203 | **< 0.001** |
|  | **kd. treatment: infection** | 7.802 | 1.094 | 7.131 | **< 0.001** |
| *TmAtt1b* | **kd. treatment** | -5.571 | 0.909 | -6.130 | **< 0.001** |
|  | **infection** | -11.971 | 1.538 | -7.783 | **< 0.001** |
|  | **kd. treatment: infection** | 4.885 | 2.175 | 2.246 | **0.025** |
| *TmAtt2* | **kd. treatment** | -3.808 | 0.7162 | -5.317 | **< 0.001** |
|  | **infection** | -9.202 | 0.7162 | -12.848 | **< 0.001** |
|  | kd. treatment: infection | 0.4937 | 1.0129 | 0.487 | 0.626 |
| *TmTen1* | **kd. treatment** | -3.7717 | 0.463 | -8.140 | **< 0.001** |
|  | **infection** | -7.7504 | 0.710 | -10.916 | **< 0.001** |
|  | **kd. treatment: infection** | 2.2323 | 1.004 | 2.223 | **0.026** |
| *TmTen2* | **kd. treatment** | -9.227 | 1.618 | -5.702 | **< 0.001** |
|  | **infection** | -12.329 | 1.618 | -7.619 | **< 0.001** |
|  | **kd. treatment: infection** | 8.011 | 2.288 | 3.501 | **< 0.001** |
| *TmTen4* | **kd. treatment** | -6.091 | 0.512 | -11.887 | **< 0.001** |
|  | **infection** | -9.943 | 0.512 | -19.407 | **< 0.001** |
|  | **kd. treatment: infection** | 5.588 | 0.725 | 7.712 | **< 0.001** |
| *TmColA* | **kd. treatment** | -5.973 | 0.941 | -6.346 | **< 0.001** |
|  | **infection** | -12.560 | 0.941 | -13.344 | **< 0.001** |
|  | **kd. treatment: infection** | 5.345 | 1.331 | 4.016 | **< 0.001** |
| *TmColB* | **kd. treatment** | -4.844 | 0.582 | -8.325 | **< 0.001** |
|  | **infection** | -10.314 | 0.582 | -17.724 | **< 0.001** |
|  | **kd. treatment: infection** | 4.131 | 0.822 | 5.020 | **< 0.001** |
| *TmCec2* | kd. treatment | -0.816 | 0.522 | -1.562 | 0.118 |
|  | **infection** | -1.525 | 0.522 | -2.918 | **0.003** |
|  | kd. treatment: infection | 0.996 | 0.739 | 1.349 | 0.177 |
| *TmDef_L* | **kd. treatment** | -3.580 | 0.647 | -5.535 | **< 0.001** |
|  | **infection** | -7.879 | 0.647 | -12.181 | **< 0.001** |
|  | kd. treatment: infection | 0.514 | 0.915 | 0.562 | 0.574 |

**Additional file 1: Table S2. Generalized linear mixed model results for the effects of gut microbiota and infection on AMP expression**. Results of a generalized linear mixed models testing the effect of presence of gut microbiota, infection and their interaction on AMP expression, separately for each AMP.

| Gene name | Predictor | estimate | SE | Z value | *p* value |
| --- | --- | --- | --- | --- | --- |
| *TmAtt1a* | **gut microbiota** | 1.754 | 0.565 | 3.103 | **0.001** |
|  | **infection** | -9.006 | 0.434 | -20.730 | **< 0.001** |
|  | gut microbiota: infection | -1.166 | 0.614 | -1.898 | 0.058 |
| *TmAtt1b* | **gut microbiota** | 3.950 | 1.921 | 2.056 | **0.040** |
|  | **infection** | -6.784 | 2.173 | -3.122 | **0.002** |
|  | **gut microbiota: infection** | -5.319 | 2.238 | -2.377 | **0.018** |
| *TmAtt2* | gut microbiota | 0.513 | 0.424 | 1.210 | 0.226 |
|  | **infection** | -9.627 | 0.424 | -22.681 | **< 0.001** |
|  | gut microbiota: infection | -0.746 | 0.600 | -1.243 | 0.214 |
| *TmTen1* | gut microbiota | 0.668 | 0.603 | 1.107 | 0.268 |
|  | **infection** | -7.890 | 0.603 | -13.083 | **< 0.001** |
|  | gut microbiota: infection | -1.620 | 0.853 | -1.899 | 0.058 |
| *TmTen2* | gut microbiota | 0.832 | 1.479 | 0.563 | 0.574 |
|  | **infection** | -6.440 | 1.512 | -4.260 | **< 0.001** |
|  | gut microbiota: infection | -0.774 | 2.054 | -0.377 | 0.706 |
| *TmTen4* | **gut microbiota** | 1.441 | 0.556 | 2.592 | **0.010** |
|  | **infection** | -9.506 | 0.556 | -17.099 | **< 0.001** |
|  | **gut microbiota: infection** | -1.736 | 0.786 | -2.208 | **0.027** |
| *TmColA* | gut microbiota | 0.718 | 0.603 | 1.191 | 0.234 |
|  | **infection** | -10.630 | 0.603 | -17.627 | **< 0.001** |
|  | gut microbiota: infection | -1.1530 | 0.853 | -1.352 | 0.176 |
| *TmColB* | **gut microbiota** | 1.293 | 0.649 | 1.991 | **0.047** |
|  | **infection** | -9.529 | 0.649 | -14.679 | **< 0.001** |
|  | **gut microbiota: infection** | -1.979 | 0.918 | -2.156 | **0.031** |
| *TmCec2* | gut microbiota | -0.623 | 0.571 | -1.089 | 0.276 |
|  | **infection** | -1.467 | 0.571 | -2.567 | **0.010** |
|  | gut microbiota: infection | 0.433 | 0.808 | 0.536 | 0.592 |
| *TmDef_L* | gut microbiota | 1.000 | 0.705 | 1.418 | 0.156 |
|  | **infection** | -7.076 | 0.913 | -7.750 | **< 0.001** |
|  | **gut microbiota: infection** | -2.926 | 0.997 | -2.935 | **0.003** |

**Additional file 1: Table S3. Primer sequences used in this study**

| **Gene Name** | **Primer sequence** |
| --- | --- |
| *TmAtt1a*_qPCR_Fw *TmAtt1a*_qPCR_Rv | 5′-GAAACGAAATGGAAGGTGGA-3′ 5′-TGCTTCGGCAGACAATACAG-3′ |
| *TmAtt1b*_qPCR_Fw *TmAtt1b*_qPCR_Rv | 5′-GAGCTGTGAATGCAGGACAA-3′ 5′-CCCTCTGATGAAACCTCCAA-3′ |
| *TmAtt2*_qPCR_Fw *TmAtt2*_qPCR_Rv | 5′-AACTGGGATATTCGCACGTC-3′ 5′-CCCTCCGAAATGTCTGTTGT-3′ |
| *TmTen1*_qPCR_Fw *TmTen1*_qPCR_Rv | 5′-CAGCTGAAGAAATCGAACAAGG-3′ 5′-CAGACCCTCTTTCCGTTACAGT-3′ |
| *TmTen2*_qPCR_Fw *TmTen2*_qPCR_Rv | 5′-CAGCAAAACGGAGGATGGTC-3′ 5′-CGTTGAAATCGTGATCTTGTCC-3′ |
| *TmTen4*_qPCR_Fw *TmTen4*_qPCR_Rv | 5′-GGACATTGAAGATCCAGGAAAG-3′ 5′-CGGTGTTCCTTATGTAGAGCTG-3′ |
| *TmColA*_qPCR_Fw *TmColA*_qPCR_Rv | 5′-GGACAGAATGGTGGATGGTC-3′ 5′-CTCCAACATTCCAGGTAGGC-3′ |
| *TmColB*_qPCR_Fw *TmColB*_qPCR_Rv | 5′-CAGCTGTTGCCCACAAGTG-3′ 5′-CTCAACGTTGGTCCTGGTGT-3′ |
| *TmCec2*_qPCR_Fw *TmCec2*_qPCR_Rv | 5′-TACTAGCAGCGCCAAAACCT-3′ 5′-CTGGAACATTAGGCGGAGAA-3′ |
| *TmDefL*_qPCR_Fw *TmDefL*_qPCR_Rv | 5′-GGGATGCCTCATGAAGATGTAG-3′ 5′-CCAATGCAAACACATTCGTC-3′ |
| *TmL27a*_qPCR_Fw *TmL27a*_qPCR_Rv | 5′-TCATCCTGAAGGCAAAGCTCCAGT-3′ 5′-AGGTTGGTTAGGCAGGCACCTTTA-3′ |
| *TmRelish*_qPCR_Fw *TmRelish*_qPCR_Rv | 5′-AGCGTCAAGTTGGAGCAGAT-3′ 5′-GTCCGGACCTCATCAAGTGT-3′ |
| *dsTmRelish*_Fw *dsTmRelish*_Rv | 5′-TAATACGACTCACTATAGGGGACGTGCACCATCAATA-3′ 5′-TAATACGACTCACTATAGGGGCGTGTTTGGCCTTGAT-3′ |
| ds*EGFP*_Fw | 5′-TAATACGACTCACTATAGGGCTTAATGCACCACCACCACCAC-3′ |
| ds*EGFP*_Rv | 5′-TAATACGACTCACTATAGGGGTGACCCAGGATGTTACCGTC-3′ |
| 515F-16srRNA | 5′-GTGYCAGCMGCCGCGGTA-3′ |
| 806R-16srRNA | 5′-GGACTACNVGGGTWTCTAAT-3′ |

**※Underline indicates T7 promoter sequences.**
