# Supplementary material for: Immune-mediated indirect interaction between gut microbiota and bacterial pathogens
Source: BMC Biol. 2025 Sep 16;23:278. doi: 10.1186/s12915-025-02399-1 (PMC12442303; doi:10.1186/s12915-025-02399-1)
Supplement: Supplementary file 2 — Additional file 2: Detailed results. A—Effect of TmRelish knockdown on gut composition in Tenebrio larvae. B—Effect of chloramphenicol on gut microbiota diversity in Tenebrio larvae. C—Tenebrio molitor Relish knockdown efficiency. [file 12915_2025_2399_MOESM2_ESM.docx]

**Additional file 2: Detailed Results**

**Additional file 2: A. Effect of *TmRelish* knockdown on gut composition in *Tenebrio* larvae**

A Bray-Curtis dissimilarity matrix analysis based on differential bacterial genus presence and abundance revealed a significant effect of the knockdown treatment on bacterial microbiota composition (R² = 0.29, F = 8.52, *p* < 0.001), (Additional file 1: Fig.S2a). Notably, the normalized counts of three operational taxonomic units (OTUs), including two assigned to the genus *Bacillus* (family *Bacillaceae 1* and *Bacillaceae 2*) and one to the genus *Pediococcus* (family *Lactobacillaceae*), were significantly reduced following *TmRelish* knockdown (adjusted *p* < 0.05) (Additional file 1: Fig. S2c–d). These findings indicate a regulatory role of *TmRelish* in maintaining a balanced gut microbial community, potentially through modulating the proliferation of specific gut bacterial taxa. 16S rRNA amplicon sequence analysis revealed that *Clostridiales*, *Entomoplasmatales*, *Lactobacillales*, *Bacillales*, and *Enterobacteriales* dominated the ds*EGFP*-treated larvae (Additional file 1: Fig.S2e). In ds*TmRelish*-treated larvae, certain bacterial orders, such as *Entomoplasmatales* (genus: *Spiroplasma*), *Enterobacteriales*, and *Lactobacillales*, were dominant, while some were absent compared to ds*EGFP*-treated controls (Additional file 1: Fig. S2f); although *Spiroplasma* showed a visually notable increase (Additional file 1: Fig. S2e), differential abundance analysis revealed this increase was not statistically significant (adjusted *p* < 0.1).

**Additional file 2: B. Effect of chloramphenicol on gut microbiota diversity in *Tenebrio* larvae**

We investigated whether disruption of bidirectional interaction via antibiotic-treated diet will affect the gut microbiota balance. Using both culture-dependent and culture-independent approaches, we assessed the gut microbiota composition of *Tenebrio* larvae in either conventionally reared (CR) or antibiotic-treated (AB). The results showed that AB-treated larvae had no culturable microbiome compared to CR-treated larvae (data not shown). Beta-diversity analysis revealed significant clustering patterns of microbial communities between AB and CR (R² = 0.21, F = 5.98, *p* < 0.001), suggesting a high degree of similarity in microbial communities within the AB group, and greater variability within the CR group (Additional file 1: Fig. S6). Analysis showed that one taxon, from the genus *Bacillus* (family *Bacillaceae*), was significantly more abundant in the CR compared to the AB group (adjusted *p* < 0.05) (Additional file 1: Fig. S6 b). Examination of 16S rRNA gene amplicon sequencing revealed that CR larvae had an increased relative abundance of operational taxonomic units (OTUs) predominantly belonging to the orders *Lactobacillales* (genus *Lactococcus*) and *Bacillales* (genus *Bacillus*), whereas AB larvae were more dominated by orders such as *Enterobacterales*, *Rhodospirillales*, *Rhizobiales*, and *Pseudomonadales* (Additional file 1: Fig. S6 c-d). Taken together, these results suggest that CR group maintained a healthy and stable gut microbiota communities, while AB group not only exhibited a significant shift but also developed a distinct gut microbiota that is either resistant to chloramphenicol or is opportunistic colonizers in a disrupted microbiome.

**Additional file 2: C. *Tenebrio molitor* Relish knockdown efficiency**

We recapitulated our previous finding, showing that *TmRelish* is efficiently knocked down compared to ds*EGFP* on the third day post-dsRNA injection (ds*EGFP*/ds*TmRelish*: t _(31)_ = 4.817, *p* < 0.001) (Additional file 1: Fig. S7a). The respective knockdown remained significantly downregulated relative to the control at seven days post-injection (ds*EGFP*/ds*TmRelish*: t _(31)_ = 3.939, *p* = 0.0004) as measured by qRT-PCR (Additional file 1: Fig. S7a). *TmRelish* is efficiently knocked down compared to ds*EGFP* on the third day post-dsRNA injection in individuals (ds*EGFP*/ds*TmRelish*: t _(10)_ = -6.861, *p* < 0.001) (Additional file 1: Fig. S7b).
